# Silica-Inspired Aerogel Thermal Metamaterials with Gradient Porosity: High-Temperature-Induced Pore Sintering Evolution via Nanoindentation

**DOI:** 10.3390/gels12080684

**Published:** 2026-08-03

**Authors:** Yiming Song, Mingyang Yang, Shuxu Li, Huiyu Yang, Ying Yin, Mu Du

**Affiliations:** 1 School of Resources Engineering, Xi’an University of Architecture and Technology, Xi’an 710055, China; songyi6420@outlook.com (Y.S.);; 2 Key Laboratory of Thermo-Fluid Science and Engineering (Xi’an Jiaotong University), Ministry of Education, Xi’an 710049, China; 3 Shandong Key Laboratory of Hydrogen Energy Equipment and Safety, College of New Energy, China University of Petroleum (East China), Qingdao 266580, China; yinyingupc@gmail.com; 4 Shenzhen Research Institute, Shandong University, Shenzhen 518057, China; 5 Institute for Advanced Technology, Shandong University, Jinan 250061, China

**Keywords:** silica-inspired aerogel, localized pressure-assisted sintering, nanoindentation, pore-network evolution, molecular dynamics

## Abstract

Localized densification of nanoporous silica under combined mechanical compression and elevated temperature involves coupled pore collapse, skeletal rearrangement, and thermally activated sintering. Clarifying how local pre-compression regulates these processes is important for understanding the surface and near-surface densification of nanoporous silica and related porous materials. In this study, the microscopic sintering behavior of a silica-inspired aerogel-like nanoporous model under the coupling of non-uniform local stress and high-temperature fields (indentation depths of 50–150 Å and temperatures of 298–1800 K) was systematically investigated using molecular dynamics simulations combined with a three-dimensional (3D) topological recognition algorithm (probe sphere method and DBSCAN clustering). The results indicate that the sintering densification of the silica-inspired aerogel model exhibits significant pore-size dependence and a “depth-temperature inverse relationship”: the local pre-compression induced by the 150 Å indentation facilitates thermally activated atomic rearrangement and shifts the onset of densification to a lower temperature, leading to an early bimodal splitting of the pore size distribution at 1300 K, accompanied by a significant jump in the elastic modulus from 3.0 to 10.07 GPa. In contrast, the 50 Å shallow region requires heating to 1800 K to achieve an equivalent densification effect. Furthermore, topological analysis quantitatively reveals the phase transition process of the pore network from connected to isolated: taking 1300 K as an example, the number of connected pore clusters decreases from the initial 86 to 70 (at 1000 ps), marking the fracture of the connected network; subsequently, the number of isolated pores surges to 4861, and the residual connected framework is severely fragmented into 136 micro-clusters. Based on the above microstructural and topological evolution data, a four-stage thermo-mechanical synergistic evolution process of the silica-inspired aerogel model is summarized. These findings provide quantitative fundamental data that conceptually supports the design of functional gradient structures with alternating “dense-thermally-conductive” and “porous-thermally-insulating” layers within a single continuous aerogel matrix; such structures may be realized in the future through strategies such as arrayed nanoindentation combined with high-temperature sintering.

## 1. Introduction

Flexible wearable electronic devices (such as electronic skin, health monitoring patches, etc.) are developing towards high power and high integration [1,2], a trend that has led to increasingly severe localized heating problems within the devices. Such devices must closely adhere to human skin during practical service [3]. However, the human body has a relatively low threshold for thermal pain sensation (typically 42–45 °C) [4]. This poses an inherent thermal management conflict for flexible electronic devices: it is necessary to rapidly dissipate the massive heat generated by the chips [5], while simultaneously strictly preventing the heat from transferring towards the skin to avoid burns [6]. Traditional homogeneous thermal management materials cannot effectively resolve the aforementioned conflict [7]. For instance, high thermal conductivity materials such as graphene or metal films, while dissipating heat, can easily transfer heat downwards and burn the skin [8]. Conversely, pure thermal insulation materials, such as conventional homogeneous aerogels, can protect the human body but lead to significant heat accumulation at the chip interface, triggering device failure [9,10,11]. Therefore, there is an urgent need in engineering applications to develop gradient or anisotropic thermal management materials [12]. Such materials are required to possess good thermal conductivity in the in-plane direction or specific regions, while effectively blocking heat flow in the thickness direction [13]. This urgent demand, which considers both device heat dissipation and human comfort, requires researchers to precisely control the heat conduction pathways by designing the microscopic spatial structure within the materials.

Faced with the complex requirements for directional heat conduction in flexible electronic devices, relying solely on altering the chemical composition or intrinsic properties of materials has become insufficient for achieving technical breakthroughs [14]. Consequently, thermal metamaterials provide a novel theoretical approach to addressing this challenge. The core design philosophy of thermal metamaterials is that structure determines the heat conduction path; specifically, precise control over heat flow distribution is realized by artificially designing specific spatial pore networks at the microscopic scale rather than relying on the intrinsic chemical properties of the material [15,16,17]. Due to their ultra-high porosity and extremely low initial thermal conductivity, nanoporous silica aerogels have become ideal substrates for constructing such thermal metamaterials [18]. Research indicates a positive correlation between the effective thermal conductivity of aerogels and their three-dimensional (3D) framework density; a localized increase in density directly expands the heat conduction cross-sectional area of the solid skeleton and enhances heat transfer efficiency [19]. Therefore, if composite structures featuring alternating dense thermally conductive frameworks and loose thermal insulating barriers can be artificially constructed within a uniform, low-thermal-conductivity aerogel, directional guidance and localized blocking of heat flow can be achieved within a single continuous medium [20]. However, when implementing this theoretical concept of customizing heat conduction pathways through localized structural densification on relatively fragile nanoporous networks, practical physical processing remains constrained by the limitations of existing micro-nano manufacturing technologies.

In the process of exploring localized densification of aerogels to customize heat conduction pathways, a profound understanding of their microscopic sintering and mechanical evolution mechanisms is a prerequisite for achieving precise processing [21]. Previous studies have extensively explored the uniform densification behavior of aerogels. Phalippou et al. [22] systematically reviewed the sintering mechanisms of aerogels and revealed the density increase and mechanical strengthening behavior caused by surface diffusion and neck growth at high temperatures. Woignier et al. [23] established the power–law scaling relationship between the fractal structure and elastic modulus of aerogels through a series of experiments, while observing an exponential increase in Young’s modulus with rising temperature during the sintering process. Yang et al. [24,25] combined experiments with molecular dynamics simulations to elucidate the evolution of nanopores at high temperatures, identifying defect regions as initial coalescence points that drive pore collapse, thereby revealing the atomic-scale mechanisms of localized pore coalescence and network disintegration. However, most of these studies are based on simplified physical assumptions of uniform thermal fields or overall uniaxial compression. For example, Yue et al. [26] simulated the high-temperature structural evolution of defective aerogels considering free sintering without external stress fields, while the systems investigated by Rawa et al. [27] regarding the temperature effect on mechanical properties were composites restricted to uniform uniaxial compression states. Current understanding based on uniform physical fields cannot provide guidance for achieving precise spatial customization of the internal structure within a single porous network under complex non-uniform stress. To address this theoretical and processing gap, this study proposes a synergistic thermo-mechanical approach combining the non-uniform localized stress field of nanoindentation with high-temperature sintering. This method aims to induce controllable physical transitions in the fragile aerogel framework by disrupting localized stress and thermodynamic equilibrium states, ultimately providing fundamental mechanistic guidance for the design of complex heat conduction networks.

The physical basis of this synergistic thermo-mechanical approach lies in the microstructural evolution induced by thermo-mechanical coupling and the subsequent changes in heat transfer behavior. During the mechanical pre-treatment stage, the localized stress field applied by nanoindentation causes the aerogel framework to reach a yield state. This mechanical action leads to localized plastic deformation and partial pore collapse in the indented region [28]. The collapse of the framework shortens the spatial distance between nanoparticles and increases the physical contact area among them [29]. In the subsequent high-temperature sintering stage, the indented and unindented regions exhibit divergent physical evolution processes. Due to the closer particle arrangement formed initially, the sintering activation energy for atomic surface diffusion in the compressed region is correspondingly reduced. This alteration in thermodynamic conditions promotes preferential interfacial fusion in this area, gradually forming sintering necks [30]. Conversely, the unindented region, which maintains a higher initial porosity and fewer particle contacts, experiences a smaller degree of framework shrinkage under identical heating conditions, thereby retaining the original nanoscale mesopores [23]. This structural differentiation, triggered by the mechanical field and further driven by the thermal field, reflects the synergistic effect of the physical processes. According to relevant heat transfer theories, the aforementioned non-uniform microstructure directly affects the thermal transport characteristics within the material [31]. The solid network within the indented region helps to increase the phonon mean free path, forming localized heat conduction pathways [32]. Meanwhile, the loose unindented region, which retains pore interfaces, continues to exert a thermal insulation effect by inducing interfacial phonon scattering [33]. Therefore, employing atomic-scale computational simulation methods to elucidate these multi-stage topological evolution rules is essential for quantitatively verifying this physical synergistic mechanism.

To clarify the atomistic mechanisms of localized pressure-assisted sintering in nanoporous silica, this study employs nanoindentation as an idealized loading approach to introduce a non-uniform local stress field and different degrees of pre-compression into the porous framework. Nanoindentation initially induces localized pore collapse and skeletal rearrangement, thereby modifying the local contact geometry and stress state. Subsequent high-temperature relaxation provides the thermal activation required for atomic diffusion, local viscous rearrangement, neck-like growth, and further densification. Therefore, the observed structural evolution is not attributed to mechanical loading alone: temperature drives the thermally activated restructuring of the silica framework, whereas indentation mainly regulates the onset and spatial distribution of these processes through local pre-compression. Molecular dynamics simulations are combined with the probe sphere method and the DBSCAN clustering algorithm to quantitatively characterize the evolution of pore size distribution and three-dimensional pore connectivity under coupled thermo-mechanical conditions. The results reveal a depth-dependent shift in the onset of densification, the emergence and disappearance of bimodal pore-size distributions, and the progressive transition of the pore network from connected to isolated states. Based on these observations, a four-stage evolution mechanism is proposed to describe the localized pressure-assisted sintering process. The present study focuses on atomistic mechanisms and relative structural evolution trends within an idealized silica-inspired aerogel-like nanoporous model, rather than on the direct fabrication or device-scale performance prediction of thermal-management components. These findings provide a mechanistic basis for future multiscale investigations of structurally graded porous materials.

## 2. Results and Discussion

### 2.1. Load–Displacement (P-h) Curves

**Figure 1:**
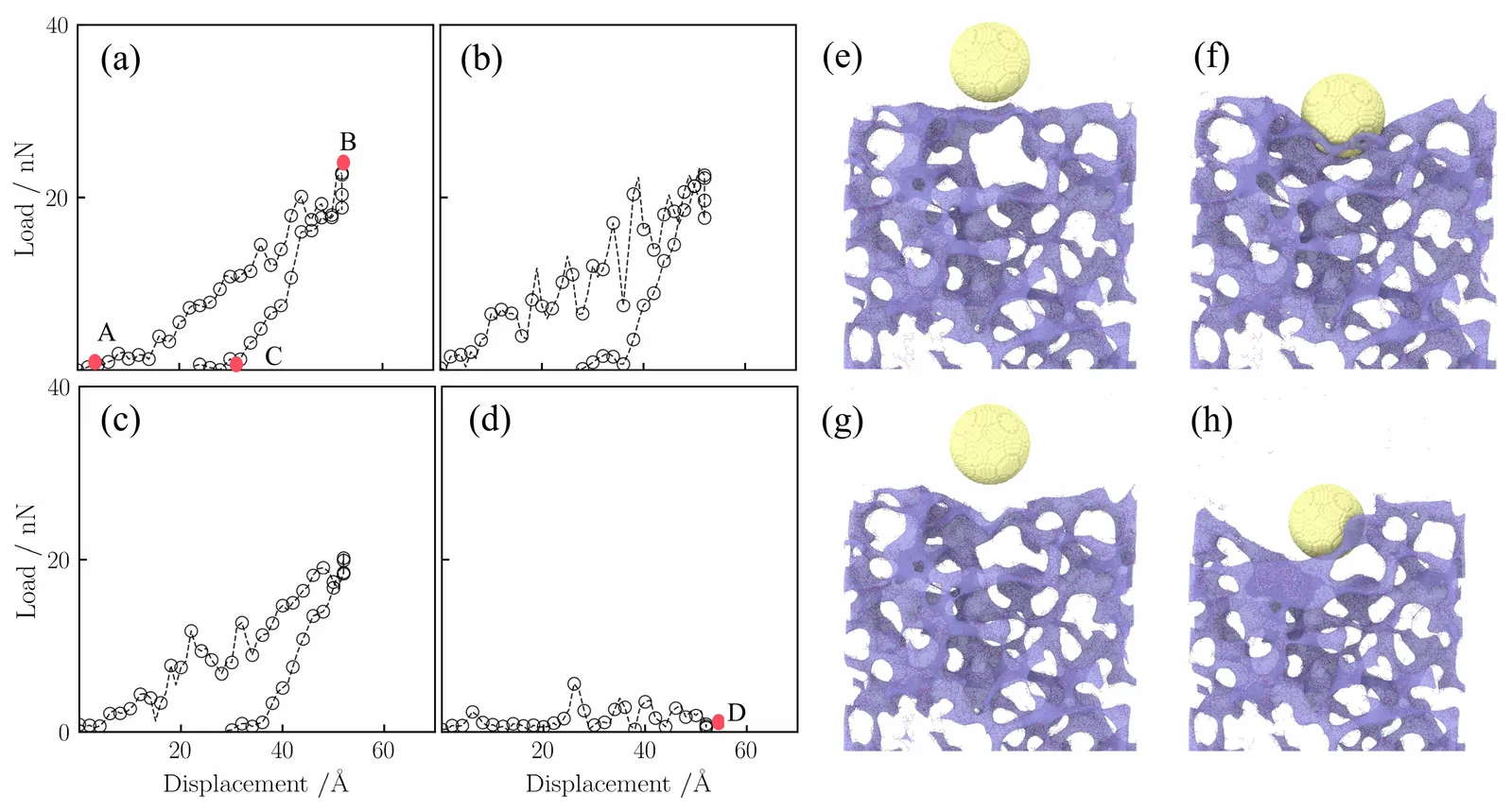
Load–displacement curves and microstructural configurations of aerogels at key nodes under different temperatures. (**a**–**d**) show the load–displacement curves at an indentation depth of 50 Å under ambient temperatures of 298 K, 973 K, 1300 K, and 1500 K, respectively. (**e**–**g**) present the atomic arrangement states at 298 K for the initial contact (node A), the maximum depth of 50 Å (node B), and the fully unloaded state (node C). (**h**) illustrates the micromorphology at the maximum depth of 50 Å (node D) under a temperature of 1500 K.

**Figure 2:**
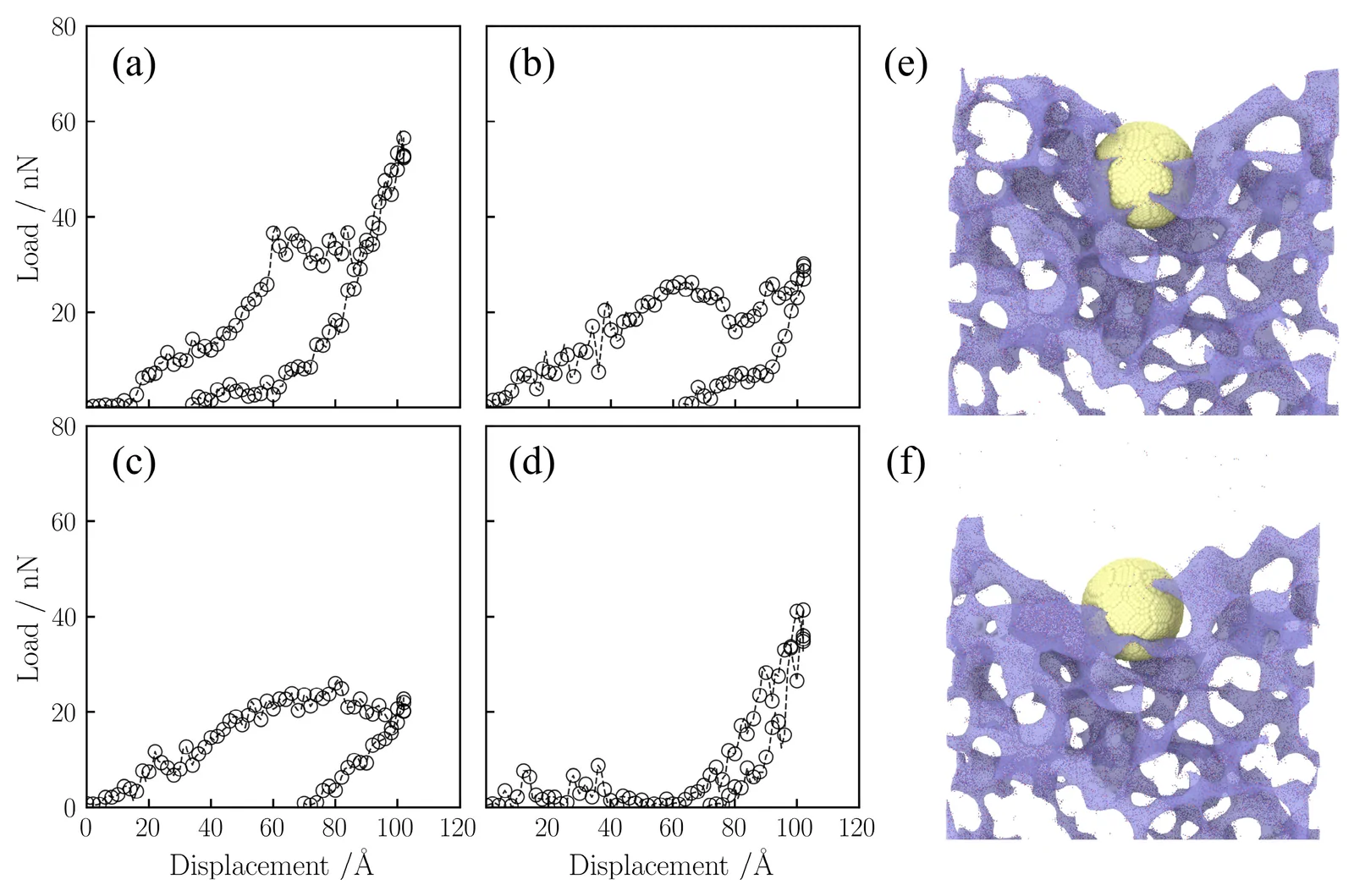
Load–displacement curves and microstructures of the silica-inspired aerogel model at an indentation depth of 100 Å. (**a**–**d**) are the load–displacement curves corresponding to 298 K, 973 K, 1300 K, and 1500 K, respectively. (**e**,**f**) are the microstructure images at the maximum depth for 298 K and 1500 K, respectively.

**Figure 3:**
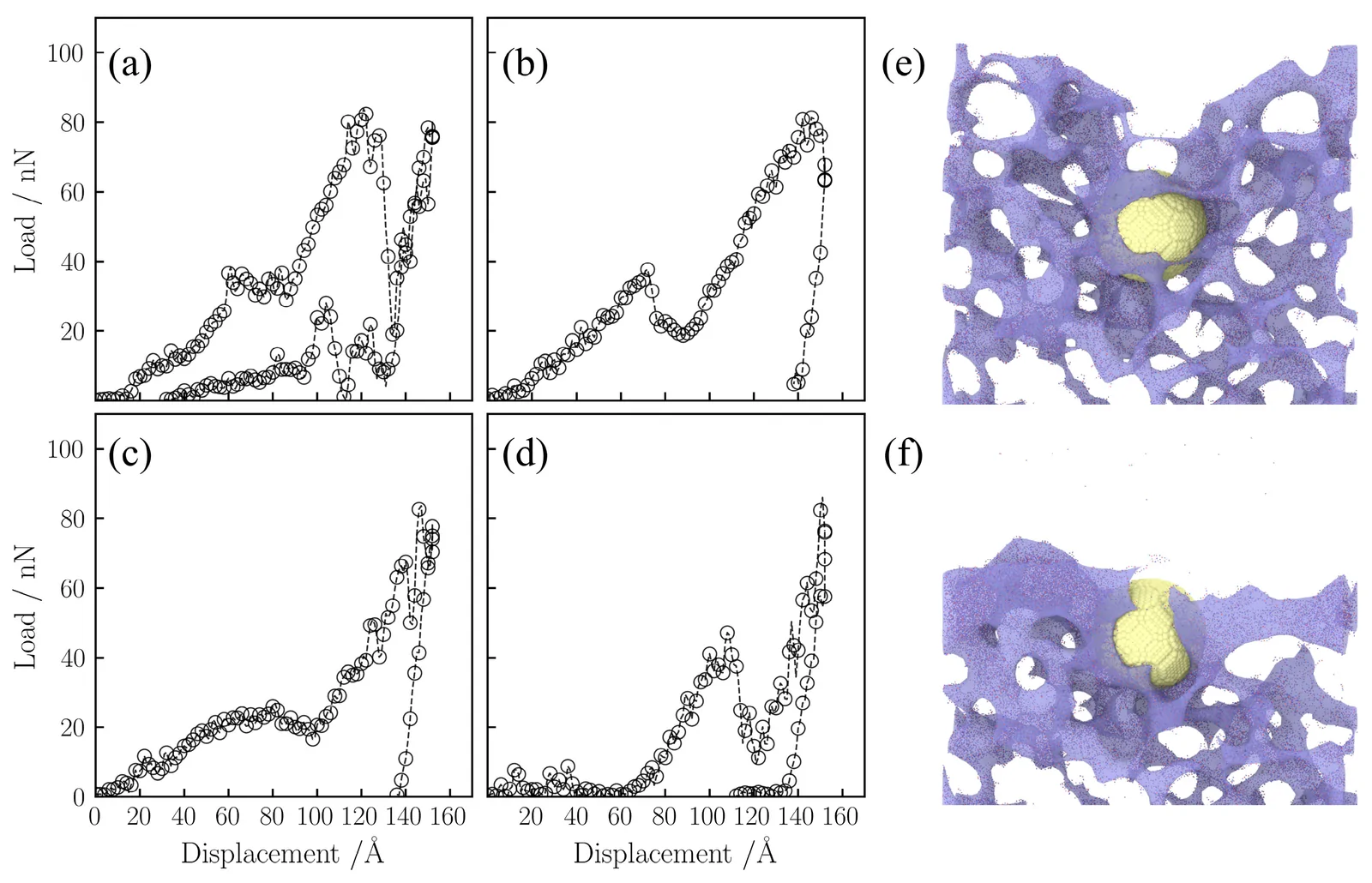
Load–displacement curves and microstructures of the silica-inspired aerogel model at an indentation depth of 150 Å. (**a**–**d**) correspond to the load–displacement curves at 298 K, 973 K, 1300 K, and 1500 K, respectively. (**e**,**f**) are the microstructure images at the maximum depth for 298 K and 1500 K, respectively.

Figure 1, Figure 2 and Figure 3 illustrate the nanoindentation load–displacement curves for three indentation depths (50, 100, and 150 Å) at temperatures of 298, 973, 1300, and 1500 K. The complete temperature evolution curves for all indentation depths (50, 80, 100, 120, and 150 Å) are provided in Supplementary Materials Figures S1–S5. Additionally, the dynamic atomistic visualizations of the full nanoindentation process at a depth of 150 Å under varying temperatures (298, 973, 1300, and 1500 K) are provided in Supplementary Videos S1–S4. Combined with the atomic spatial configurations at corresponding data nodes, this section visually demonstrates and analyzes the objective effects of indentation depth and high ambient temperatures on the mechanical load-bearing capacity and microscopic framework deformation characteristics of the silica-inspired aerogel model.

Figure 1 displays the load–displacement curves and microstructural evolution during the nanoindentation process. Figure 1a–d sequentially present the mechanical curves for an indentation depth of 50 Å at temperatures of 298, 973, 1300, and 1500. At 298 K, the force increases nonlinearly with indenter penetration, and the first load drop occurs at 18 Å, where the force decreases from 4.1 nN to 2.2 nN. The subsequent loading phase is accompanied by continuous serrated fluctuations, reaching a peak load of 23.4 nN at the maximum depth of 50 Å. The curve upon unloading to zero indicates a residual indentation depth of 27 Å. As the system temperature rises to 973 K, the overall peak load increases to 29.9 nN and occurs at a depth of 38 Å. When the temperature further increases to 1300 K and 1500 K, the peak loads sharply attenuate to 21.2 nN and 13.2 nN, respectively. Notably, at 1500 K, the peak occurs prematurely at 13 Å, and the load drops to only 0.3 nN at the maximum depth of 50 Å. Figure 1e–g correspond to the atomic configurations at 298 K for the initial contact (node A), the maximum depth of 50 Å (node B), and the fully unloaded state (node C), respectively. Figure 1h corresponds to node D at 50 Å depth at 1500 K; compared with the room-temperature state at the same depth (Figure 1f), the silica framework at 1500 K exhibits a more extensive deformation region and physical separation from the bottom of the indenter, which visually explains the extremely low local resistance of only 0.3 nN at this point.

Figure 2 presents the load–displacement curves and the microstructural configurations at the maximum depth for an indentation depth of 100 Å. The peak load at 298 K is 60.23 nN. As the system enters the high-temperature regime, the peak loads at 973 K and 1300 K are significantly attenuated by 49% and 56% compared to the maximum at 298 K, reaching 31.05 nN and 27.04 nN, respectively. However, at an extreme high temperature of 1500 K, the peak load anomalously climbs by 51% compared to that at 1300 K, reaching 40.75 nN. Figure 2e illustrates the localized high-density silica region formed directly beneath the indenter at 298 K. Figure 2f shows that at 1500 K, the softened framework undergoes plastic flow and closely packs to envelop the indenter; these microscopic densification features visually explain the phenomenon where the load-bearing capacity on the 1500 K curve abnormally increases by 51%.

Figure 3 illustrates the load–displacement curves and the microstructural configurations at the maximum indentation depth for an indentation depth of 150 Å at 298 K and 1500 K. At 298 K and 973 K, the peak loads at an indentation depth of 150 Å are 84.00 nN and 82.59 nN, respectively. As the ambient temperature rises to 1300 K and 1500 K, the peak loads at this depth do not attenuate but instead increase to 90.54 nN and 87.66 nN, respectively. Figure 3e presents the localized high-density region formed directly beneath the indenter at 298 K for an indentation of 150 Å. Figure 3f shows the microscopic spatial morphology at 1500 K, where the silica framework network undergoes plastic deformation and adheres to and envelops the bottom of the indenter. This dense packing configuration provides direct microstructural support for the phenomenon where the peak load data at high temperatures of 1300 K and 1500 K are higher than those at room temperature.

### 2.2. Elastic Modulus and Hardness and Their Temperature Evolution

**Figure 4:**
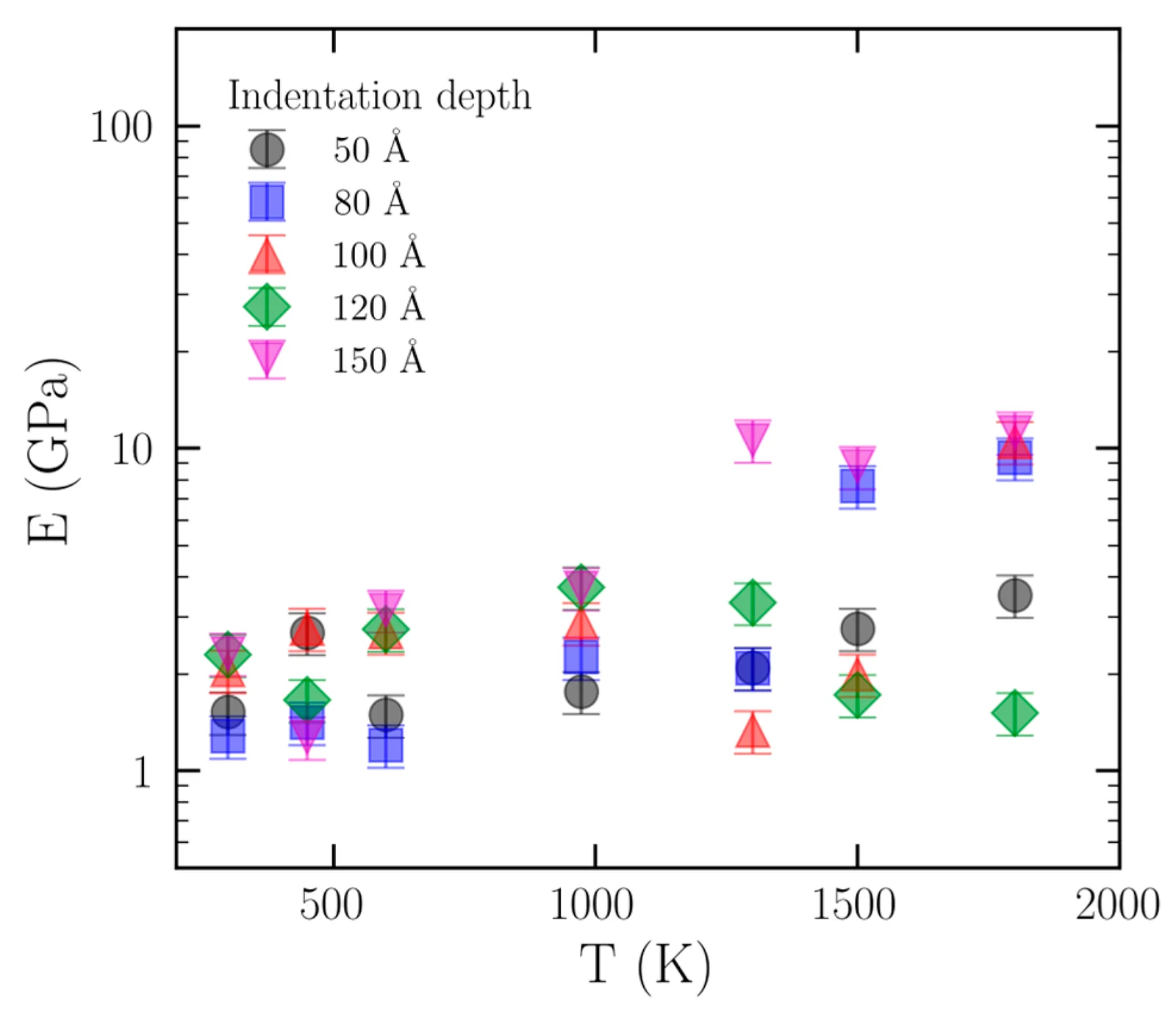
Variation in elastic modulus E as a function of temperature under different indentation depths.

**Figure 5:**
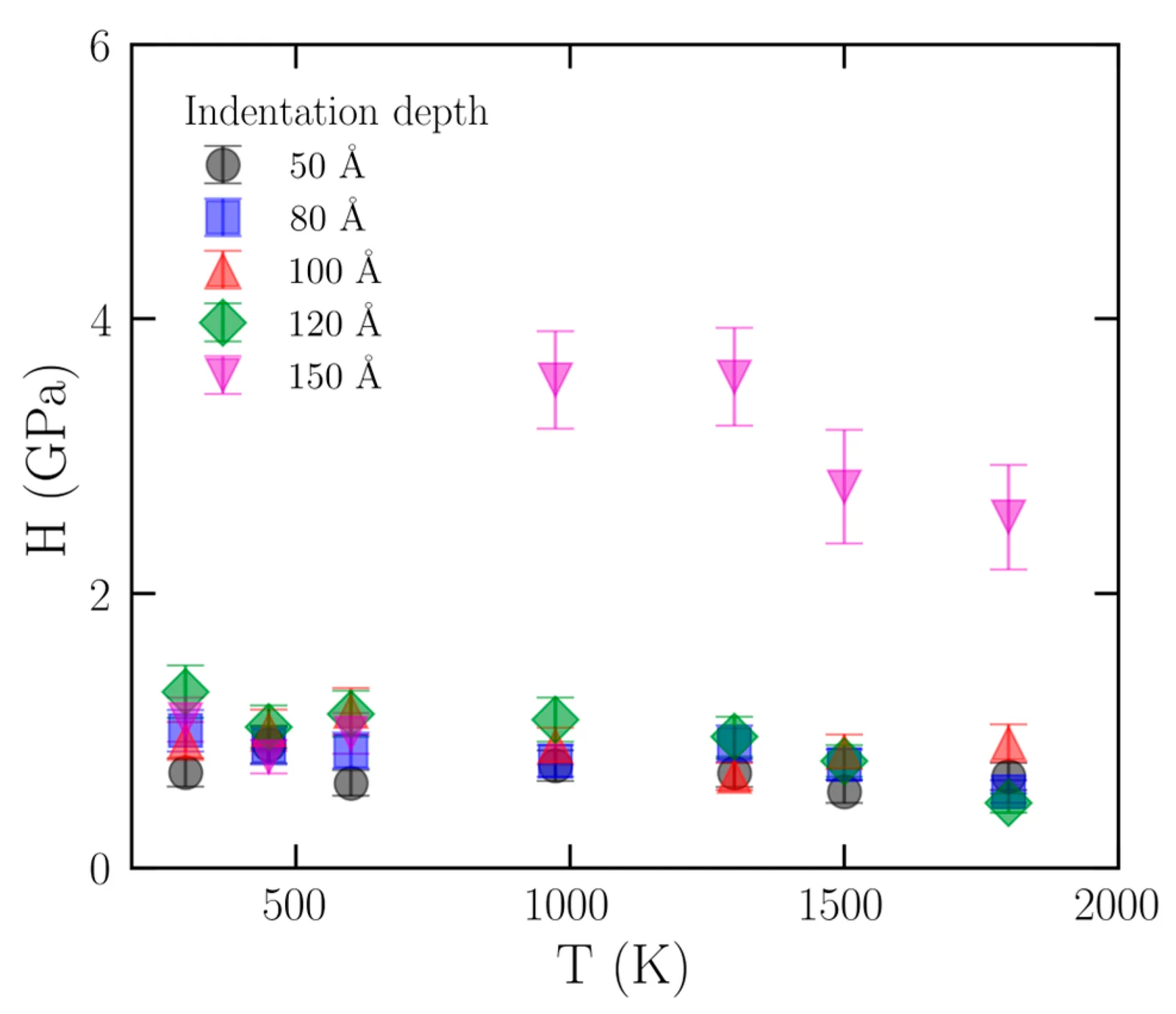
Variation in hardness H as a function of temperature under different indentation depths.

The elastic modulus (E) and hardness (H) of the silica-inspired aerogel model were calculated using the Oliver–Pharr method [34] from the unloading segment of the nanoindentation load–displacement curves for various indentation depths (50–150 Å) and temperatures (298–1800 K). The calculation results for a total of 35 independent cases are summarized in Figure 4 and Figure 5, respectively. These values are interpreted as the apparent/local effective modulus and hardness of the indented region, rather than bulk macroscopic properties.

As shown in Figure 4, the temperature dependence of the elastic modulus exhibited differences across different indentation depths. In the temperature range of 298–973 K, the E values for each indentation depth remained relatively stable: 1.4–2.5 GPa for 50 Å, 1.1–2.0 GPa for 80 Å, 1.9–2.7 GPa for 100 Å, 1.6–3.5 GPa for 120 Å, and 1.2–3.0 GPa for 150 Å. Within the temperature range of 1300–1800 K, the elastic modulus for certain depths showed a significant increase. Specifically, the E value for the 80 Å depth increased from 1.97 GPa to 7.26 GPa at 1500 K, an increase of approximately 268%. For the 100 Å depth, it increased from 1.89 GPa to 9.94 GPa at 1800 K, an increase of approximately 426%. For the 150 Å depth, the E value increased from 3.0 GPa to 10.07 GPa at 1300 K, an increase of approximately 236%, and further reached 10.64 GPa at 1800 K. The E values for the 120 Å depth remained within the range of 1.4–3.1 GPa in the corresponding temperature interval. The data indicate that the temperature thresholds at which the E values begin to rise, varying with depth: the increase for 150 Å occurs at 1300 K, while the rise for 80 Å and 100 Å occurs between 1500 and 1800 K.

The variation in hardness H with temperature in Figure 5 exhibited similar segmented characteristics. In the range of 298–600 K, the hardness values for all depths ranged from 0.5 to 1.15 GPa. As the temperature further increased, the H value for the 150 Å depth increased from 0.88 GPa to 3.20 GPa at 973 K, an increase of approximately 264%, and reached 3.22 GPa at 1300 K. At temperatures of 1500–1800 K, the actual indentation depth data for the 50 Å depth decreased to 4–11 Å. Comparing the data for the 150 Å depth in Figure 4 and Figure 5, the onset of the increase in H (973 K) occurred earlier than the onset of the increase in E (1300 K).

Combining the data from Figure 4 and Figure 5, the temperature dependence of the elastic modulus and hardness of the silica-inspired aerogel model can be categorized into three regimes: in the 298–600 K regime, the mechanical indicators for all depths remain relatively stable; in the 973–1300 K regime, the values for the 150 Å depth begin to rise; and in the 1300–1800 K regime, values for other depths also undergo significant increases, with the temperature thresholds for the increase showing a correspondence with the indentation depth.

### 2.3. Model Validation

**Figure 6:**
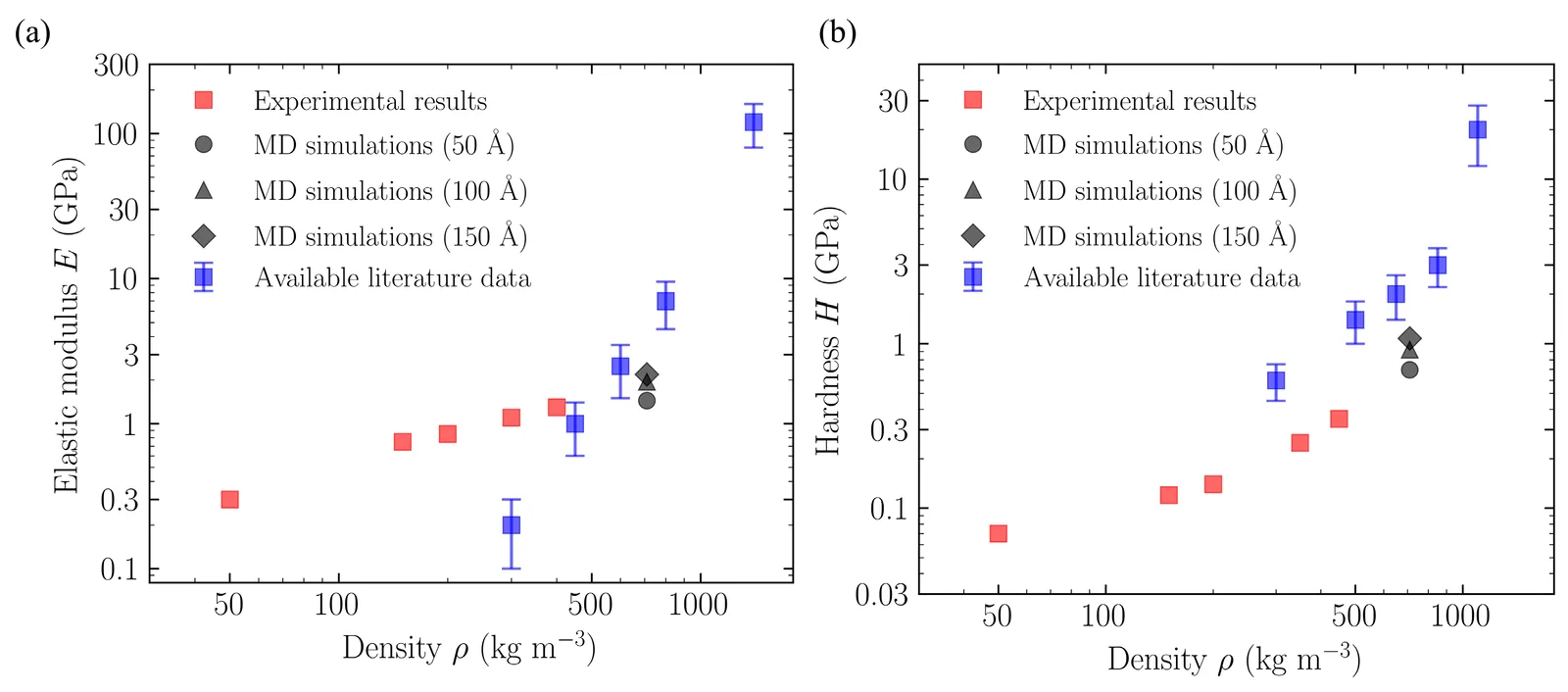
Model validation: (**a**) Validation of the elastic modulus-density relationship; (**b**) Validation of the hardness–density relationship.

To ensure the reliability of the molecular dynamics (MD) simulation results obtained in this study, the calculated elastic modulus and hardness were systematically validated against experimental measurements and previously reported simulation data from the literature. Based on the Oliver–Pharr method, the contact stiffness was extracted from the unloading segment of the nanoindentation curves, from which the E and H were subsequently determined. Figure 6 presents the variation in elastic modulus and hardness as a function of density. Through quantitative comparison with existing literature data, the physical plausibility and comparability of the local mechanical response are assessed.

Figure 6a shows the relationship between elastic modulus E and density ρ. The figure includes three types of comparative data: experimental data based on macroscopic mechanical testing (50–400 kg/m^3^, E=0.3–1.3 GPa) [35,36,37,38,39], previous MD numerical simulation data from the literature (300–1400 kg/m^3^, E=0.2–120 GPa) [40,41,42,43,44], and the MD simulation data from this study (710 kg/m^3^). In log-log coordinates, the elastic modulus and density exhibit a typical power–law relationship ($E\propto \rho^{n},\;n\approx 3–4$). At 298 K, the elastic modulus values for the five indentation depths in this study are as follows: 1.44 GPa at 50 Å, 1.21 GPa at 80 Å, 1.95 GPa at 100 Å, 2.17 GPa at 120 Å, and 2.19 GPa at 150 Å, with an average value of 1.79±0.42 GPa. The MD simulation data points in this study fall on the power–law trend line reported in the literature and are in good agreement with the elastic modulus distribution range for aerogels of the same density reported by Patil et al. [34]. This comparison confirms the comparability and physical plausibility of the local effective modulus and hardness extracted here, and serves as the first step of the micro-to-macro bridging, rather than a claim that the model predicts bulk macroscopic properties.

Figure 6b shows the relationship between hardness H and density ρ. The experimental data [34] cover the low-density region (50–450 kg/m^3^, H=0.07–0.35 GPa), while the literature data [45] cover the medium-to-high density region (300–1100 kg/m^3^, H=0.6–20 GPa). The MD simulations in this study show that at 298 K, the hardness increases with the indentation depth: 0.70 GPa at 50 Å, 0.84 GPa at 80 Å, 0.92 GPa at 100 Å, 0.97 GPa at 120 Å, and 1.08 GPa at 150 Å, exhibiting an indentation size effect (ISE). This trend is consistent with the hardness–density relationship for nanoporous silica aerogels reported in the literature [34]. Combining the validation results from Figure 6a,b, the elastic modulus and hardness obtained from the MD simulations in this study are consistent with the literature experimental data in terms of quantitative trends.

### 2.4. Effect of Temperature on Pore Size Distribution

**Figure 7:**
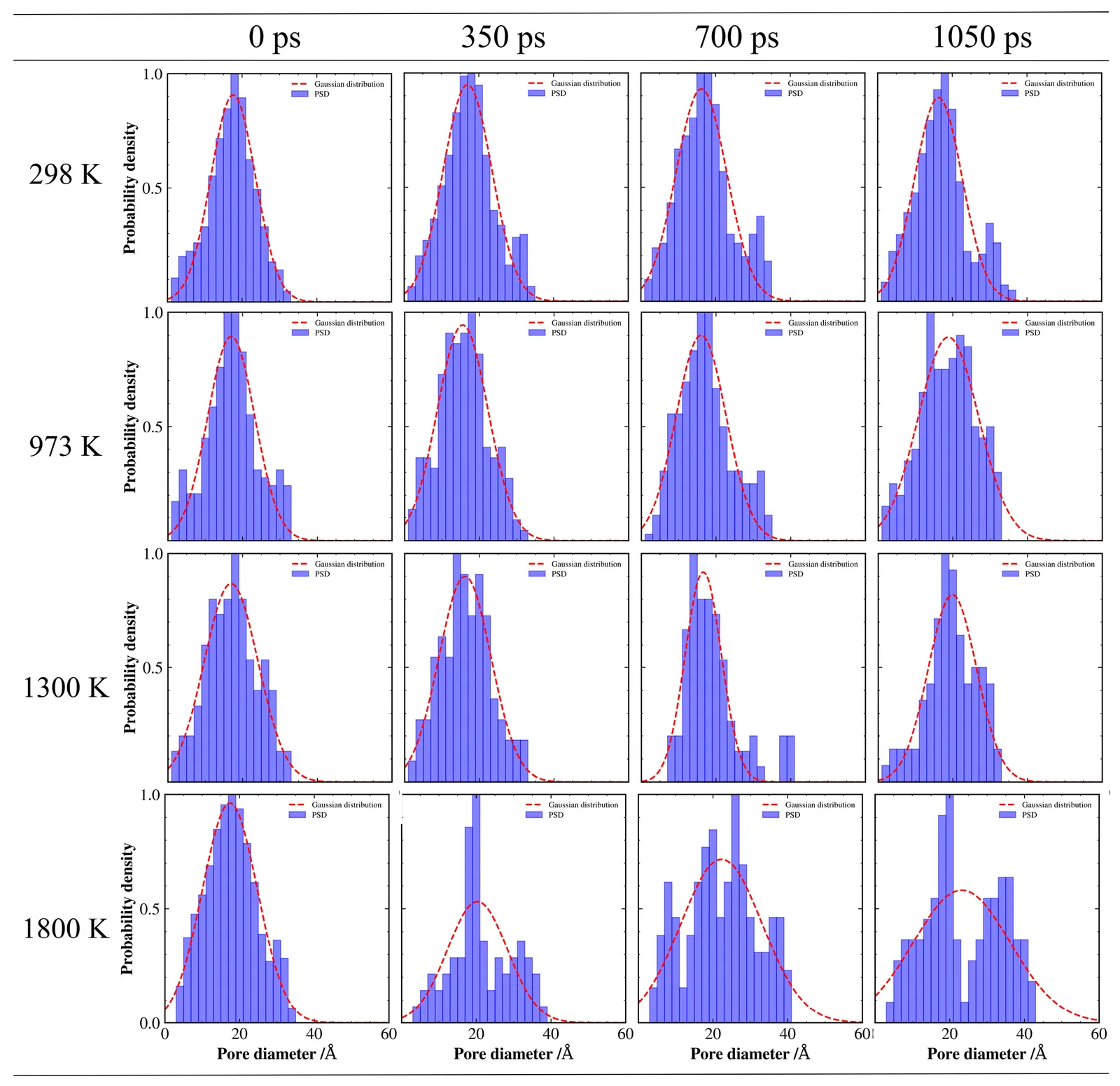
Evolution of pore size distribution at different temperatures and time points under an indentation depth of 150 Å.

**Figure 8:**
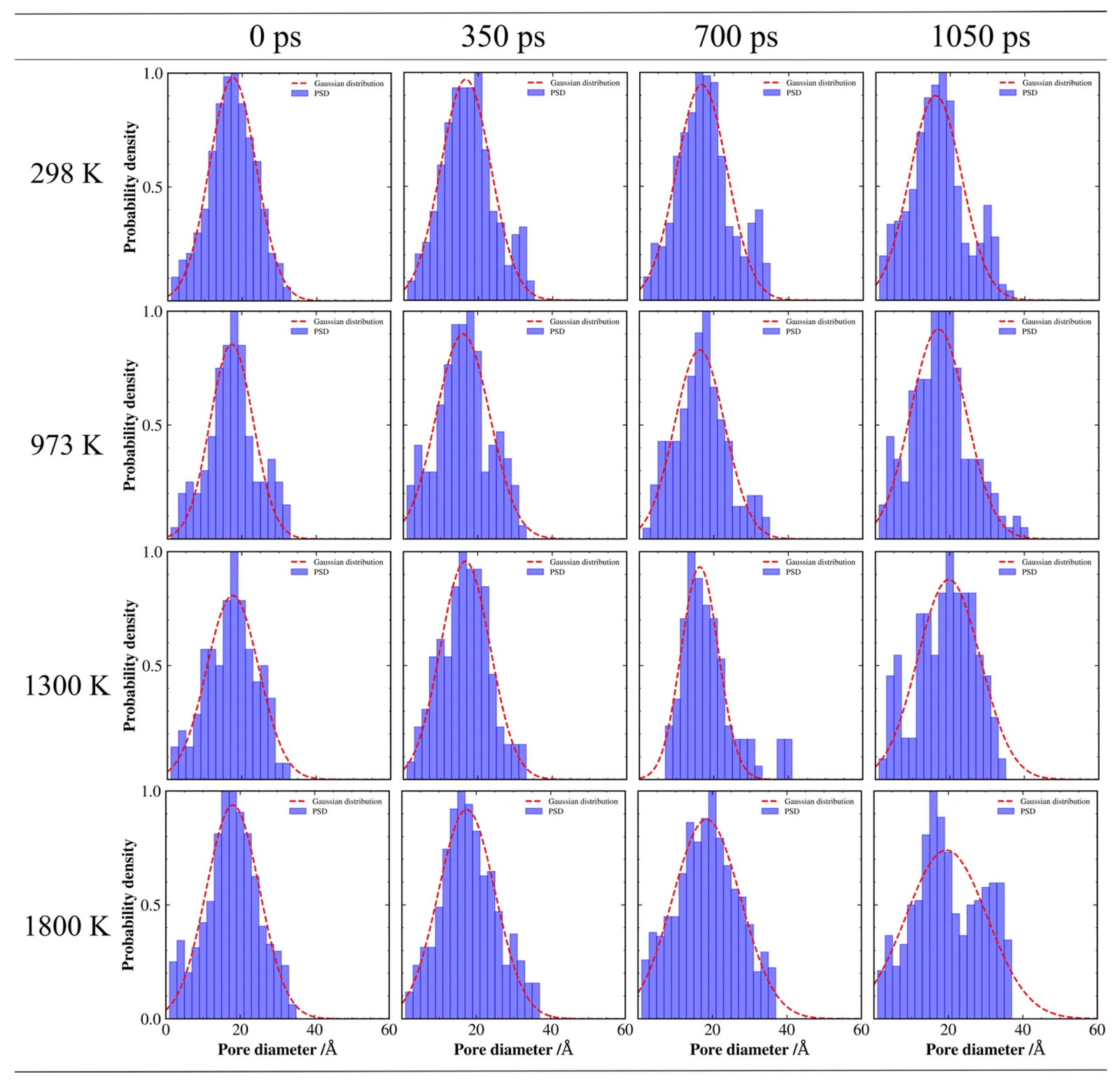
Evolution of pore size distribution at different temperatures and time points under an indentation depth of 100 Å.

**Figure 9:**
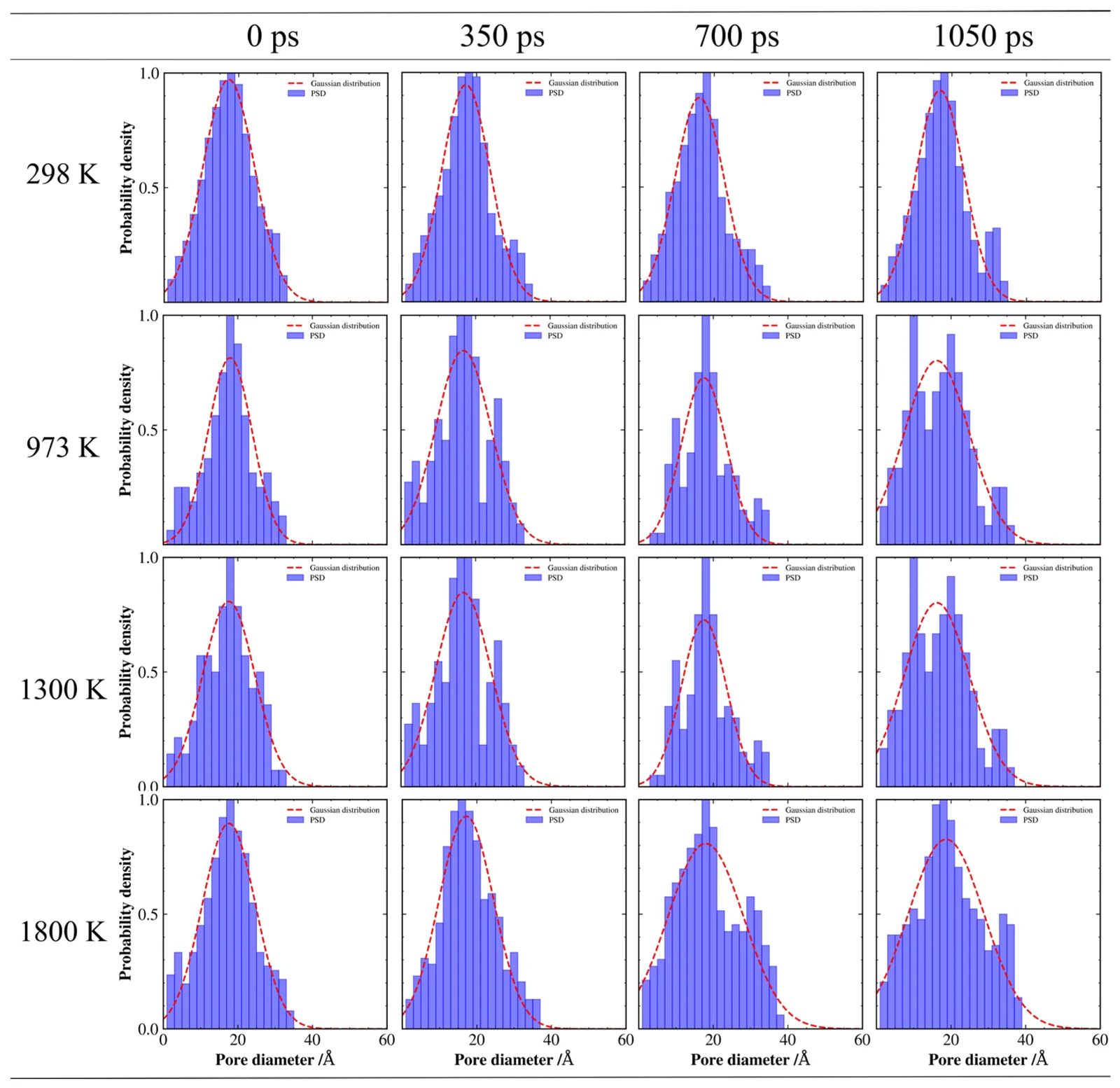
Evolution of pore size distribution at different temperatures and time points under an indentation depth of 50 Å.

To reveal the pore evolution patterns during the sintering process of the silica-inspired aerogel model, this section provides an in-depth analysis of the temperature response characteristics of the pore structure at different indentation depths based on the PSD data extracted from nanoindentation simulations. Figure 7, Figure 8 and Figure 9 present the evolution patterns of the PSD for the silica-inspired aerogel model at indentation depths of 150 Å, 100 Å, and 50 Å across a temperature range of 298–1800 K. Detailed pore size evolution data for all simulated depths are provided in Supplementary Materials Figures S6–S10.

Figure 7 illustrates the microscopic evolution characteristics of the silica-inspired aerogel model PSD with respect to temperature and time at an indentation depth of 150 Å. At an ambient temperature of 298 K, the pore structure remains thermodynamically stable, with the primary pore size peak concentrated around 20 Å, a peak probability density of approximately 0.09, and a distribution of large pores extending to the right. When the system temperature increases to 973 K and the time relaxes to 1050 ps, the primary peak shifts leftward to approximately 17 Å, and the peak probability density drops below 0.07; simultaneously, the probability density in regions larger than 40 Å increases significantly compared to the initial state. This leftward shift of the primary peak and the broadening of the large-pore region mark the sintering precursors of initial necking and coalescence of large pores driven by surface energy. As the temperature further rises to 1300 K, the evolution curves at 700 ps and 1050 ps exhibit a distinct bimodal splitting phenomenon, with the primary peak shrinking to approximately 15 Å and a secondary large-pore peak forming in the 35–45 Å range. This bimodal topological configuration indicates that network rearrangement occurs under the coupling of high temperature and deep localized stress, with some large pores undergoing local fusion while tiny pores collapse under pressure. At a high temperature of 1800 K and a relaxation time of 1050 ps, the secondary large-pore peak above 35 Å completely disappears, and the probability density of the small-pore peak in the 15–18 Å range increases significantly to over 0.15. The aforementioned topological evolution sequence, from the initial coalescence of large pores to bimodal splitting and finally to small-pore focusing, objectively depicts the continuous physical process of the aerogel framework evolving from local fusion strengthening to overall densification.

Mechanistically, the leftward shift of the primary peak reflects surface-energy-driven atomic diffusion toward concave neck regions between adjacent particles, leading to neck growth and partial coalescence of large pores [22]. The subsequent bimodal splitting arises because the collapsed large pores leave behind residual voids in the 35–45 Å range while the pressure simultaneously squeezes smaller pores to below 20 Å. This sequence is consistent with the viscous-sintering picture of silica aerogels, where the full pore-size distribution evolves through neck growth, coalescence, and final densification [22], and with the three-stage sintering scenario summarized in recent reviews. These structural changes should not be interpreted as the result of mechanical compaction alone. High temperature provides the thermal activation required for surface diffusion, local viscous rearrangement, and neck-like growth, whereas nanoindentation mainly modifies the initial contact geometry and local stress state through pre-compression. The earlier structural evolution observed at greater indentation depths is therefore interpreted as localized pressure-assisted thermal sintering rather than purely mechanical pore collapse.

Comparing the pore size evolution sequences across the three depths in Figure 7, Figure 8 and Figure 9 clearly reveals the regulatory role of indentation depth on the sintering process. At 973 K, the deep region at 150 Å already exhibits significant distribution broadening and a full width at half maximum (FWHM) of approximately 25 Å, while the distributions at shallow-to-medium depths of 100 Å and 50 Å remain in a low-temperature state with FWHMs of approximately 18–20 Å and primary peaks stable around 20 Å. This difference stems from the non-uniform stress field gradient induced by nanoindentation; the deep material sustains local pressures in the range of 3–5 GPa, and the resulting pre-compressed configuration facilitates thermally activated atomic rearrangement, causing the characteristic pore-structure evolution to occur approximately 200–300 K earlier than in the shallow region. When the ambient temperature reaches 1300 K, the distribution at a depth of 150 Å exhibits a bimodal structure at 700 ps and 1050 ps, with the primary peak at 15–17 Å and a secondary peak at 35–40 Å. This bimodal splitting reveals that in order to reduce total surface energy under the coupling of high temperature and high stress, atoms preferentially undergo surface diffusion toward high-curvature neck regions between pores, leading to localized necking and coalescence of some connected large pores, while tiny pores are squeezed during framework rearrangement [46]. Combined with the data in Section 2.2, the jump in the elastic modulus at 150 Å from 3.0 GPa to 10.07 GPa at 1300 K is consistent with the destruction of pore connectivity; as large pores are segmented into isolated holes, the load transfer path shifts from network support to dense framework support, appearing as macroscopically as a sudden increase in stiffness. This temporal difference indicates that, under the action of a non-uniform stress field, the deep region directly beneath the indenter undergoes stress-induced sintering first, resulting in the localized formation of a densification gradient structure that is “dense inside and loose outside” within the indentation area.

When the temperature further rises to the extreme of 1800 K, the PSDs at all depths evolve into multi-peak discrete structures, suggesting that sintering has entered its later stages. Taking the 150 Å depth as an example, at 1050 ps, the large-pore peak above 35 Å almost disappears, and the probability density of small pores in the 15–20 Å range increases to over 0.15. In contrast, at a depth of 50 Å, although the primary peak shifts leftward to 15 Å, the FWHM remains approximately 20 Å, and no significant secondary large-pore peak appears. The characteristic of focused small pores but remaining broadening in the deep region indicates that while bulk densification is complete, residual isolated large pores are difficult to eliminate due to being surrounded by a dense framework. At 1800 K and 150 Å depth, the elastic modulus reaches 10.64 GPa, but the increase in hardness flattens out to 3.22 GPa, indicating that the mechanical response of the material has shifted from pore-controlled deformation to framework-dominated deformation [47]. From a mechanistic perspective, the presence of large pores provides preferential atomic transport channels, thereby lowering the temperature threshold for local sintering, while the presence of a large number of tiny pores delays the overall densification process by increasing the curvature barrier for atomic rearrangement. The indentation depth regulates the activation threshold of these processes by altering the local stress state; the pre-compressed state formed by the deep high-stress region eventually facilitates the early initiation of the microscopic ultimate restructuring of the aerogel.

### 2.5. Effect of Temperature on Pore Connectivity

The temperature dependence of the pore topological structure is key to understanding the sintering mechanism. By tracking the evolution of the number of connected pore clusters and isolated pore clusters, the patterns of temperature-induced pore network topological transitions can be quantitatively identified. Taking the 150 Å depth as an example, this section analyzes the dynamic evolution characteristics of pore clusters within the temperature range of 298–1800 K and performs a horizontal comparison with the 50 Å depth to reveal the synergistic regulatory effect of temperature and indentation depth on pore topological stability. Connectivity evolution data over time for intermediate depths (80, 100, 120 Å) are provided in Supplementary Materials Figures S11–S13. Furthermore, the pore connectivity evolution quantitatively tracked based on the DBSCAN clustering algorithm is visually confirmed by three-dimensional (3D) spatial topological visualization. The distribution of characteristic positions for different topological types of pores (connected pores and isolated pores) and their dynamic succession in quantity over time have been completely recorded via video (see Supplementary Videos S5 and S6). This dynamic trajectory intuitively reflects the decay of macroscopic connected regions and the aggregation of localized isolated pores, further confirming the physical authenticity and algorithmic accuracy of the topological identification data in this section, providing a solid validation foundation for subsequent mechanistic analysis.

**Figure 10:**
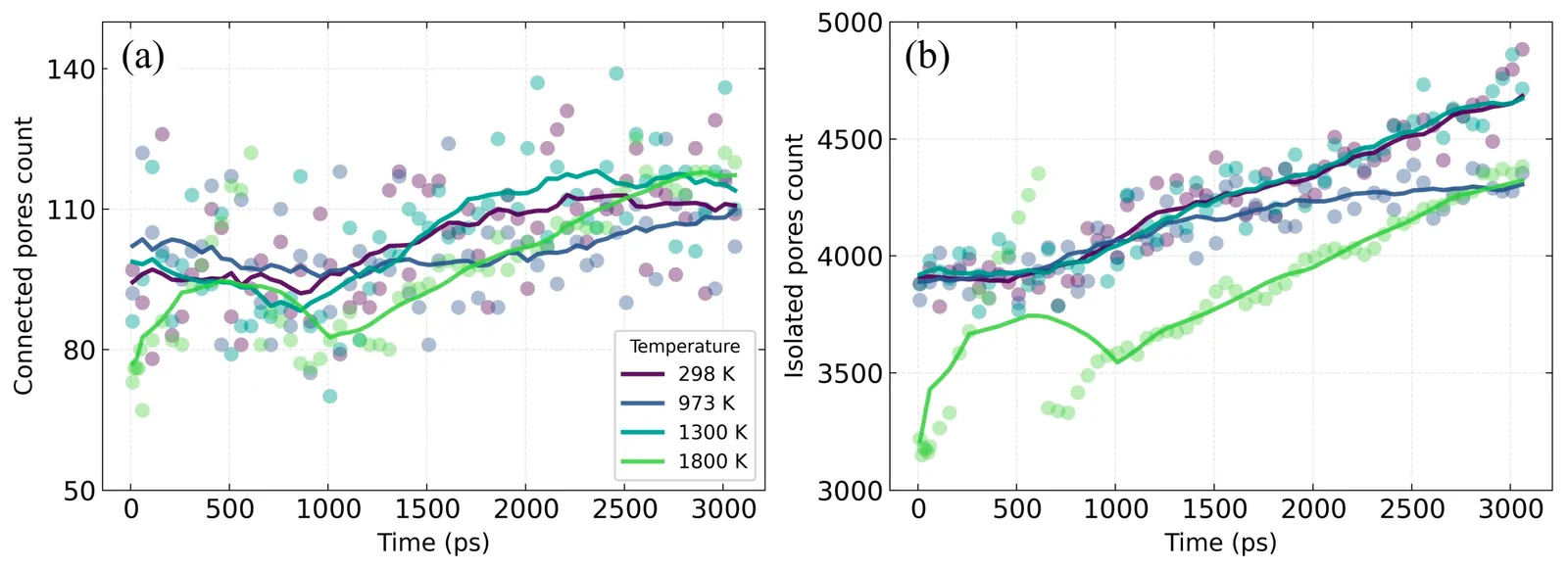
Evolution of pore connectivity over time under an indentation depth of 150 Å. (**a**) Evolution of the number of connected pore clusters; (**b**) Evolution of the number of isolated pore clusters.

Under the conditions of 298 K and 973 K, the distribution of pore clusters at a depth of 150 Å remains relatively stable (Figure 10). The number of connected pore clusters stays at approximately 100 with small fluctuations; the baseline for isolated pore clusters is larger, increasing slowly from approximately 3900 to approximately 4300. This evolution pattern indicates that while thermal vibrations in the low-to-medium temperature range can drive localized atomic surface relaxation, they are insufficient to destroy the connected pore network of the pore network. The stability of the connected pore cluster ratio corresponds to the fundamental stability of the material’s mechanical structure in this temperature range at the microscopic topological level.

The onset of the connected pore-cluster fracture near 1300 K is not a random topological fluctuation but the direct consequence of the structural reorganization shown in Figure 7. As large pores coalesce and the solid fraction locally increases, the high-curvature necks that previously bridged adjacent pores can no longer be sustained; they break and transform into isolated pore clusters. This topological transition changes the load-bearing mechanism: the material is no longer supported by a percolating pore network but by a densified solid skeleton, which manifests macroscopically as the simultaneous jump in apparent modulus and hardness seen in Figure 4 and Figure 5. The observed modulus increase is consistent with the well-known power–law scaling between elastic modulus and density in aerogels [39], while the underlying microstructural driver is neck growth and fusion between silica particles, which strengthens the solid backbone [22].

When the temperature rises to 1300 K, a significant transition occurs in the evolution of the pore cluster distribution. The number of connected pore clusters begins to show a downward trend, decreasing from approximately 100 to approximately 80; meanwhile, the number of isolated pore clusters increases significantly, rising from approximately 3900 to approximately 4800. This is consistent with as the heat-activated surface atomic diffusion intensifies, some thinner connected pore channels (i.e., high-curvature framework necks) fracture and gradually transform into isolated pore clusters. In this research system, the significant decrease in the number of connected pore clusters near 1300 K can serve as a topological reference node for judging the onset of aerogel sintering [46]. Under the high-temperature conditions of 1500 K and 1800 K, the topological evolution of the pore network is even more pronounced. Taking 1800 K as an example, the number of isolated pore clusters rapidly increases to over 4500, and the number of connected pore clusters shows distinct localized fluctuations at approximately 700 ps. This trend reflects that extreme high temperatures significantly accelerate surface atomic diffusion and the reconstruction process of pore boundaries. Severe structural densification causes some pores to be compressed or even undergo localized boundary fusion, leading to brief structural adjustments in the connectivity indicators of the pore network amidst an overall downward trend.

**Figure 11:**
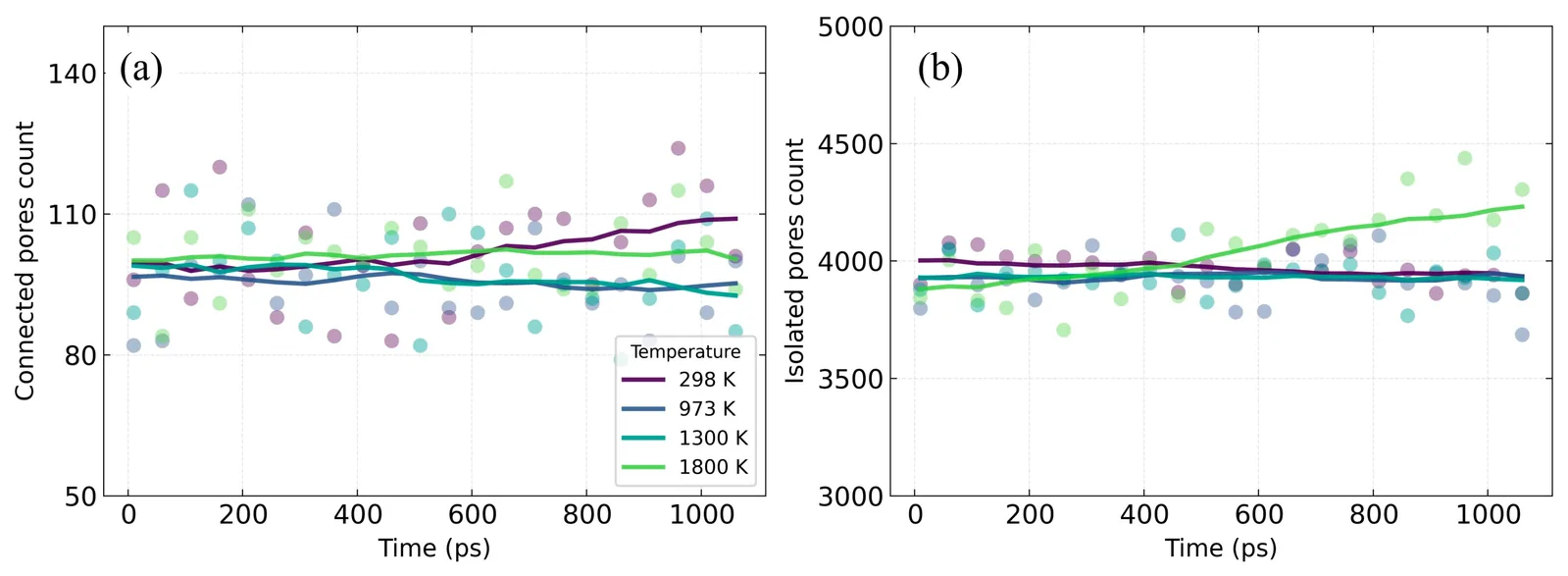
Evolution of pore connectivity over time under an indentation depth of 50 Å. (**a**) Evolution of the number of connected pore clusters; (**b**) Evolution of the number of isolated pore clusters.

Figure 11 illustrates the evolution trajectory at a depth of 50 Å, showing an overall trend similar to that of 150 Å but with a smaller transition magnitude. At 1800 K, the increase in isolated pore clusters at 50 Å depth is significantly lower than that at 150 Å depth, and no significant fluctuations similar to those in the deep region at 700 ps are observed for the connected pore clusters. Comparison results suggest that the deep region (150 Å) is in a high-stress field dominated by the indenter, where the coupling of indentation stress and high temperature further promotes atomic diffusion and pore network reconstruction. Overall, the rise in temperature dominates the transformation from connected pore clusters to isolated pore clusters, while the deep structure exhibits relatively higher sensitivity to topological transition due to the superimposed effect of stress.

### 2.6. Influence of Indentation Depth on Pore Connectivity

**Figure 12:**
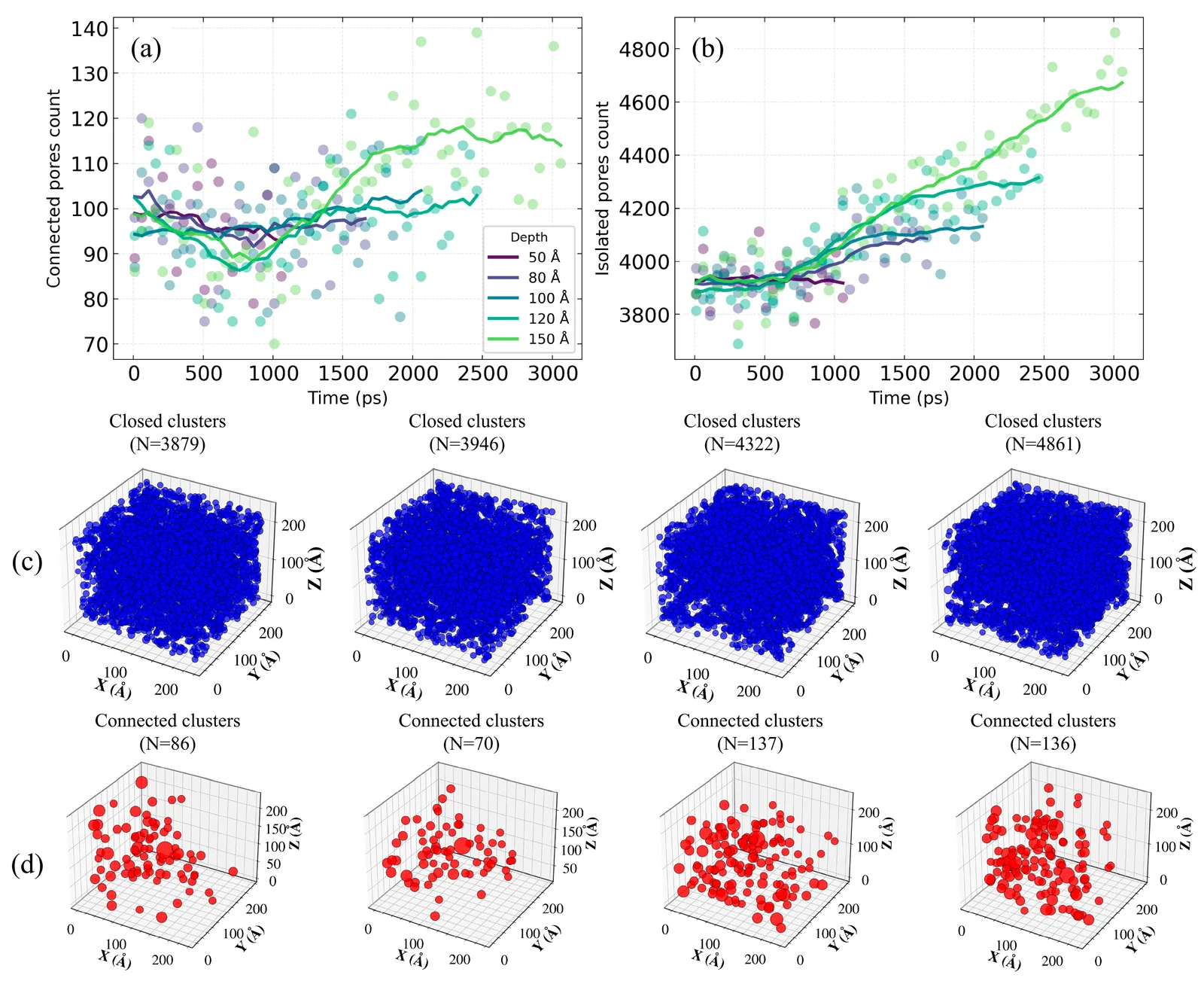
Multi-depth comparison of pore connectivity and spatial topological evolution at 1300 K. (**a**) Evolution of the number of connected pore clusters over time; (**b**) Evolution of the number of isolated pore clusters over time; (**c**) 3D spatial distribution of isolated pore clusters at typical evolution moments under an indentation depth of 150 Å; (**d**) Spatial distribution of connected pore clusters at corresponding moments under an indentation depth of 150 Å.

To further quantify the regulatory role of the stress field on pore network reconstruction, this section compares the dynamic evolution characteristics of pore connectivity across different indentation depths at the same temperature. Figure 12 illustrates the variation patterns of connected pore clusters and isolated pore clusters as a function of indentation depth at 1300 K and 1800 K, respectively. The complete multi-depth comparisons for all investigated temperatures (298, 450, 600, 973, 1500, and 1800 K) are provided in Supplementary Materials Figures S14–S19.

Data show that connected pore clusters maintain a fluctuation range of approximately 90–110 at all depths, but the fluctuation amplitude in the deep layer (150 Å) is significantly larger than that in the shallow layer (50 Å). In the shallow layer (50 Å), the mean value of connected pore clusters is approximately 97 with a standard deviation of 9.7, remaining relatively stable overall; in the middle layer (100 Å), the mean is approximately 98 with a standard deviation of 9.8; in the deep layer (150 Å), the mean rises to approximately 105 and the standard deviation increases to 14.9, indicating that the connectivity of the deep pore network is in a more unstable state. The evolutionary differences in isolated pore clusters are more significant: the shallow layer (50 Å) maintains a range of approximately 3870–4030, with a mean of 3931 and a standard deviation of only 80; the middle layer (100 Å) shows a mean increase to 4031 and a standard deviation increase to 120; the deep layer (150 Å) continuously increases from an initial value of approximately 3879 to approximately 4714, with a mean of 4247 and a standard deviation as high as 284. At a depth of 150 Å, isolated pore clusters continue to show an upward trend after approximately 2000 ps, while the 50 Å depth tends to stabilize during the same period. This critical difference indicates that the deep structure undergoes more intense pore network reconstruction in the sintering-sensitive temperature zone, where the deep indentation stress promotes the transition from connected pores to isolated pores [48].

The aforementioned depth-related evolutionary differences can be attributed to stress variations induced by the indentation depth. During the nanoindentation process, a greater indentation depth leads to higher accumulated stress within the material. At a temperature of 1300 K, the higher stress environment in the deep layer (150 Å) facilitates atomic rearrangement and enhances atomic mobility at pore boundaries, leading to the fracture of neck structures in connected pores and the formation of more isolated pore clusters. In the shallow layer (50 Å), the stress level is lower, the rate of atomic rearrangement is slower, and the pore topological structure remains relatively stable. The combined effect of the deep high-stress environment and high temperature accelerates the reconstruction process of the pore network. This result indicates that the indentation depth is a crucial factor in regulating the timing of pore sintering: the deep structure exhibits higher transition sensitivity due to the higher stress level, entering the sintering state earlier at the same temperature.

To more intuitively illustrate the destruction of the pore network topological structure in the deep high-stress zone, Figure 12c,d extract the three-dimensional (3D) spatial distribution of pore clusters at a depth of 150 Å. (the corresponding time-resolved dynamic evolution is presented in Supplementary Videos S5 and S6). When the system evolves from the initial state to the physical inflection point at 1000 ps, the number of connected pore clusters drops from 86 to 70, marking the initial fracture of the macroscopically connected network. Subsequently, during the accelerated densification phase from 2000 ps to 3000 ps, the number of isolated pores climbs sharply to 4861 and highly aggregates beneath the indenter; meanwhile, the number of connected pore clusters anomalously increases to 136. This fragmentation characteristic indicates that extreme localized stress leads to severe topological fragmentation of the remaining connected framework. This spatial evolution image is highly consistent with the aforementioned quantitative trends, visually confirming the spatial acceleration effect of thermo-mechanical synergy on the sintering and densification of aerogels.

### 2.7. Mechanism of Localized Pressure-Assisted Sintering

**Figure 13:**
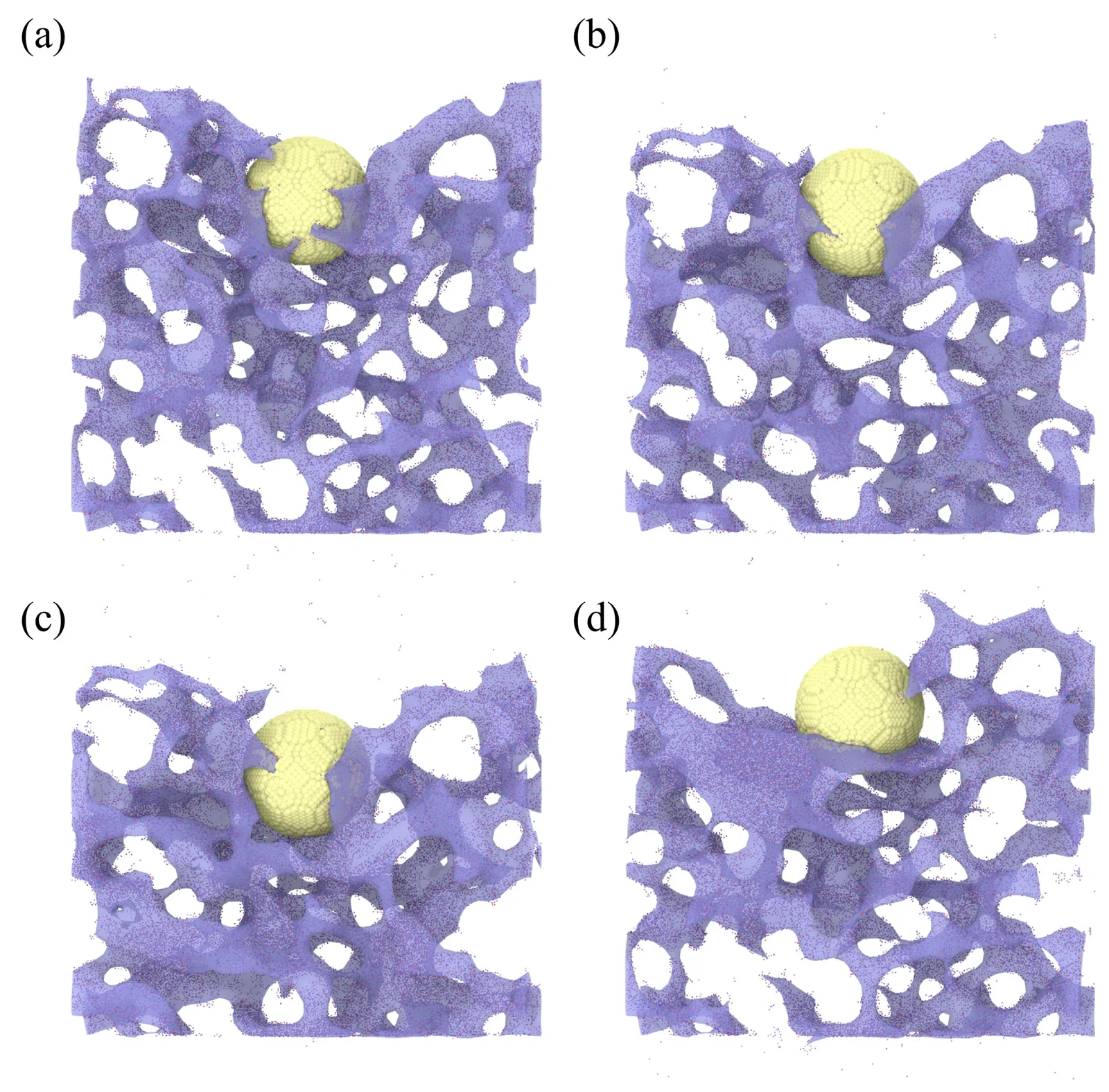
Visualization of pore topological evolution at an indentation depth of 150 Å (1000 ps). (**a**) 298 K; (**b**) 973 K; (**c**) 1300 K; (**d**) 1800 K.

Based on the analysis in Section 2.4, Section 2.5 and Section 2.6, the sintering process of the silica-inspired aerogel model can be jointly characterized by three dimensions: pore size, temperature, and indentation depth. Pore size determines sintering sensitivity, with large pores being the first to participate in structural evolution; temperature drives the topological transition of pores, promoting the transformation from connected pore clusters to isolated pore clusters; indentation depth regulates the transformation timing through stress effects, with deep structures exhibiting higher transformation activity. Figure 13 illustrates the pore structure evolution at a depth of 150 Å and a time of 1000 ps under four typical temperature conditions, visually presenting a continuous transition from loose connectivity to significant densification. At 298 K (Figure 13a), the pore network exhibits a highly connected fluffy structure, with connected pores forming continuous channels. At 973 K (Figure 13b), the pore network begins to shrink, and signs of localized densification appear, though the overall connected framework remains intact. At 1300 K (Figure 13c), the pore volume decreases significantly and the network connectivity drops markedly, marking the arrival of the connectivity fracture stage. At 1800 K (Figure 13d), structural densification is significant, with most pores having collapsed and only a small amount of isolated pore clusters remaining. Based on these structural characteristics, the sintering process can be divided into four stages [49]: the pre-heating stability stage (<973 K), the preferential coalescence of large pores stage (973–1300 K), the connectivity network fracture stage (1300–1800 K), and the limit densification stage (>1800 K). Among them, the preferential coalescence of large pores stage (973–1300 K) possesses the optimal process controllability, with a wide temperature window and a moderate structural transformation rate. Combined with the depth-temperature coupling relationship in Figure 7 and Figure 9, it can be seen that a 150 Å deep indentation can achieve significant densification at 1300 K (with the elastic modulus increasing from 3.0 GPa to 10.07 GPa), whereas a 50 Å shallow indentation requires heating to 1800 K to achieve the same effect. This depth–temperature relationship demonstrates that local pre-compression regulates the onset of thermally activated sintering. Greater indentation depths produce a more strongly pre-compressed local configuration, allowing pore-network reconstruction and densification to occur at lower temperatures [22]. The result therefore provides mechanistic evidence for the spatial localization of pressure-assisted sintering, rather than direct evidence for the fabrication of macroscopic gradient thermal structures.

This thermo-mechanical synergistic evolution mechanism provides atomistic insight into localized pressure-assisted sintering and surface densification in nanoporous silica. The high-stress zone beneath the indenter enters the sintering state earlier and forms a locally densified region, whereas the peripheral low-stress region retains more of the original porous structure. This structural contrast demonstrates how local pre-compression regulates the onset and spatial distribution of thermally activated pore-network reconstruction. In the present study, nanoindentation is therefore employed as an idealized method for generating a localized non-uniform stress field, rather than as a demonstrated manufacturing route for constructing complex thermal conduction pathways throughout bulk aerogel monoliths. Related studies nevertheless indicate that localized energy input or mechanical compression can induce spatially selective densification in porous materials, as demonstrated by laser-written and thermally vitrified silica aerogels [50] and nanoindentation-induced densification of nanoporous metals [51]. Engineered porosity gradients can also be realized in aerogels through additive manufacturing or templating methods, such as freeze-casting-assisted printing [52] and 3D nanomaterial patterning [53], while copper–PDMS thermal metamaterials have experimentally demonstrated heat-flow cloaking, focusing, and reversal [54]. Effective-medium approximations further provide a theoretical framework for estimating the thermal-conductivity contrast between dense and porous regions [19,31]. However, because the indentation-affected volume is confined primarily to the surface and near-surface region, more direct engineering approaches, such as multilayer architectures or surface coatings, may be more practical for controlling heat transport at the macroscopic scale. Therefore, the main contribution of the present study is the clarification of the microscopic structural evolution associated with localized pressure-assisted sintering, while the manufacturability, structural continuity, residual stress, long-term thermal-cycling stability, and cracking resistance of macroscopic gradient structures require further experimental investigation.

For low-temperature flexible thermal-management applications, polymeric aerogels may offer a more practical materials platform than silica aerogels requiring high-temperature processing. Polyurea aerogels are of particular interest because their density and porosity can be continuously tailored during synthesis, allowing gradient structures to be introduced without relying on localized post-processing. Their structural flexibility and density-dependent thermal transport properties also make them potentially suitable for moderate-temperature service conditions [45,55]. The mechanistic insights gained in this study may also prove valuable for understanding pore collapse, structural rearrangement, and densification in such organic aerogels under localized thermo-mechanical loading, for example, as thermal barriers between cells in lithium-ion batteries [56].

## 3. Conclusions

In this study, molecular dynamics simulations were employed to investigate the sintering behavior and microstructural evolution of a silica-inspired aerogel-like nanoporous model under coupled thermo-mechanical fields (indentation depth 50–150 Å, temperature 298–1800 K). Based on the quantitative analysis of pore size distribution, pore connectivity, and mechanical properties, the conclusions are as follows:

The microscopic sintering of the silica-inspired aerogel model exhibits distinct temperature staging and pore-size dependence. Under localized stress, the pore size regulates the evolutionary sequence of sintering: large pores (>30 Å) undergo preferential localized necking and coalescence, with bimodal splitting characteristics first appearing in the range of 973–1300 K; meanwhile, tiny pores are gradually squeezed during framework rearrangement. Accompanied by the coalescence of large pores and localized structural reconfiguration, the hardness and elastic modulus of the 150 Å deep region significantly increase at 973 K and 1300 K, respectively (with the elastic modulus rising from 3.0 GPa to 10.07 GPa), consistent with the local mechanical response to microscopic pore evolution.

Temperature elevation is the primary thermodynamic factor driving the topological transition of the pore network. Near 1300 K, the microscopic connectivity of the aerogel undergoes a significant change, with the number of connected pore clusters decreasing from approximately 100 to around 80, while high-curvature connected necks fracture and transform into isolated pores, increasing the number of isolated pore clusters to approximately 4800. This topological transition from connected to isolated states marks the destruction of the continuity of the network backbone and serves as a quantitative reference node for determining the onset of aerogel network sintering.

The non-uniform localized stress field induced by indentation depth regulates the sequence and spatial distribution of sintering. The pre-compressed configuration in the deep region facilitates thermally activated atomic rearrangement, allowing pronounced densification to occur at approximately 1300 K, whereas the shallow region requires heating to approximately 1800 K to achieve a comparable structural state. Therefore, the observed evolution is not attributed to mechanical loading alone: temperature provides the primary thermal activation for atomic diffusion, local viscous rearrangement, and neck-like growth, while indentation advances and spatially localizes these processes through pre-compression.

Based on the evolution of pore size distribution and connectivity, the localized pressure-assisted sintering process can be divided into four stages: pre-heating stability, preferential structural rearrangement, pore-network fragmentation, and limiting densification. In this study, nanoindentation is employed as an idealized means of generating local pre-compression and a non-uniform stress field, rather than as a demonstrated manufacturing strategy for macroscopic thermal-management components. The results therefore provide atomistic insight into how mechanical pre-compression modifies the onset and spatial distribution of high-temperature sintering and surface densification in the silica-inspired aerogel model. The transferability of these mechanisms to macroscopic aerogel monoliths requires further experimental and multiscale investigation.

## 4. Materials and Methods

### 4.1. Setup of Molecular Dynamics Simulations

The molecular dynamics simulations in this study were performed using LAMMPS (version 29 August 2024). The atomic-resolution nanoporous silica aerogel models were generated through a melt-quenching protocol. The interactions between silicon and oxygen atoms within the silica network were described using the Tersoff potential [57]. After equilibration and relaxation, the silica aerogel model contains 192,000 atoms, with an overall density of 710 kg/m^3^ and a porosity of approximately 67.7%. The initial spatial dimensions of the aerogel matrix were 230 Å × 230 Å × 230 Å. To accommodate the upper indenter and prevent physical interference from periodic boundaries, the simulation box was extended to 530 Å along the *Z*-axis to reserve a vacuum region of approximately 300 Å. The non-bonded interactions between the rigid indenter and the silica matrix were calculated using the Lennard–Jones potential with a cutoff radius of 10 Å. In the overall mechanical testing system, a 15 Å thick region at the bottom of the aerogel was designated as a fixed layer to prevent rigid translation during the simulation process [58]. The remainder of the matrix was set as a thermostatic layer, and the system temperature was controlled under the NVT ensemble using the Nose-Hoover thermostat algorithm. The simulation testing matrix covered seven temperature points ranging from 298 K to 1800 K and five target indentation depths ranging from 50 Å to 150 Å [25]. All combinations of set temperatures and depths were executed independently, constituting 35 complete sets of molecular dynamics simulation tests. It should be noted that the present study is an atomistic mechanistic study rather than a device-scale predictive one. The 230 Å model containing approximately 192,000 atoms at 67.7% porosity is treated as an idealized local computational volume that contains sufficient pores, skeletal connections, and curved framework regions for the present mechanistic analysis. The extracted evolution sequences and relative thresholds are statistically meaningful at the mechanistic level [24]. Since silica sintering is a thermally activated process occurring over timescales much longer than those accessible to conventional molecular dynamics simulations, elevated temperatures are employed to accelerate atomic diffusion and structural relaxation within the ps–ns simulation window. In particular, 1800 K is used to observe late-stage pore-network reconstruction and limiting densification trends, rather than to represent a directly transferable experimental temperature–time condition. Therefore, the present analysis focuses on relative structural evolution sequences and depth-dependent transition thresholds instead of absolute sintering rates. Consequently, the simulations are not intended to predict the macroscopic thermal or mechanical behavior of a complete wearable device; instead, they provide local mechanistic insight and parameter windows that can be upscaled through effective-medium or finite-element modeling in subsequent multiscale studies [25].

A detailed list of the simulation parameters and their sources is provided in Table 1. The Si–O interactions are described by the Tersoff potential, which has been widely used in molecular dynamics studies of amorphous silica, including high-temperature structure and mechanics [57]. The non-bonded interactions between the rigid spherical indenter and the silica matrix are described by the Lennard–Jones potential, with energy and size parameters taken from the Lorentz–Berthelot mixing rules following prior molecular dynamics nanoindentation studies of silica aerogels [59]. The model density of 710 kg/m^3^ corresponds to a medium-density aerogel, which lies within the range reported in both experimental and simulation studies [35,36,37,38,39,41,42,43,44]. The 230 Å cubic model containing approximately 192,000 silica atoms is treated as a representative volume element for capturing the local pore and skeletal topology, and similar model scales have been used in large-scale molecular dynamics studies of aerogel structure and mechanics [43,44]. The temperature range of 298–1800 K covers the evolution from room-temperature stability to deep sintering, consistent with previous high-temperature sintering molecular dynamics studies [25]. The indentation rate of 0.1 Å/ps is a commonly used approximation in molecular dynamics nanoindentation, and the spherical rigid indenter follows standard nanoindentation setups [34].

It should be noted that experimentally prepared silica aerogels generally exhibit multiscale fractal networks formed by silica nanoparticles and their aggregates. The melt-quenched structure adopted here does not aim to reproduce the complete hierarchical fractal morphology of real silica aerogels from the nanometer to micrometer scales. Instead, it should be treated as a hypothetical silica-inspired model that borrows the chemical and physical properties of silica while adopting an aerogel-like nanoporous network comprising interconnected pores, skeletal junctions, curved framework segments, and locally constricted regions. The model is therefore used to investigate the relative evolution of pore collapse, skeletal rearrangement, local neck-like growth, pore-network fragmentation, and densification under identical thermo-mechanical conditions.

### 4.2. Nanoindentation Simulation Settings

**Figure 14:**
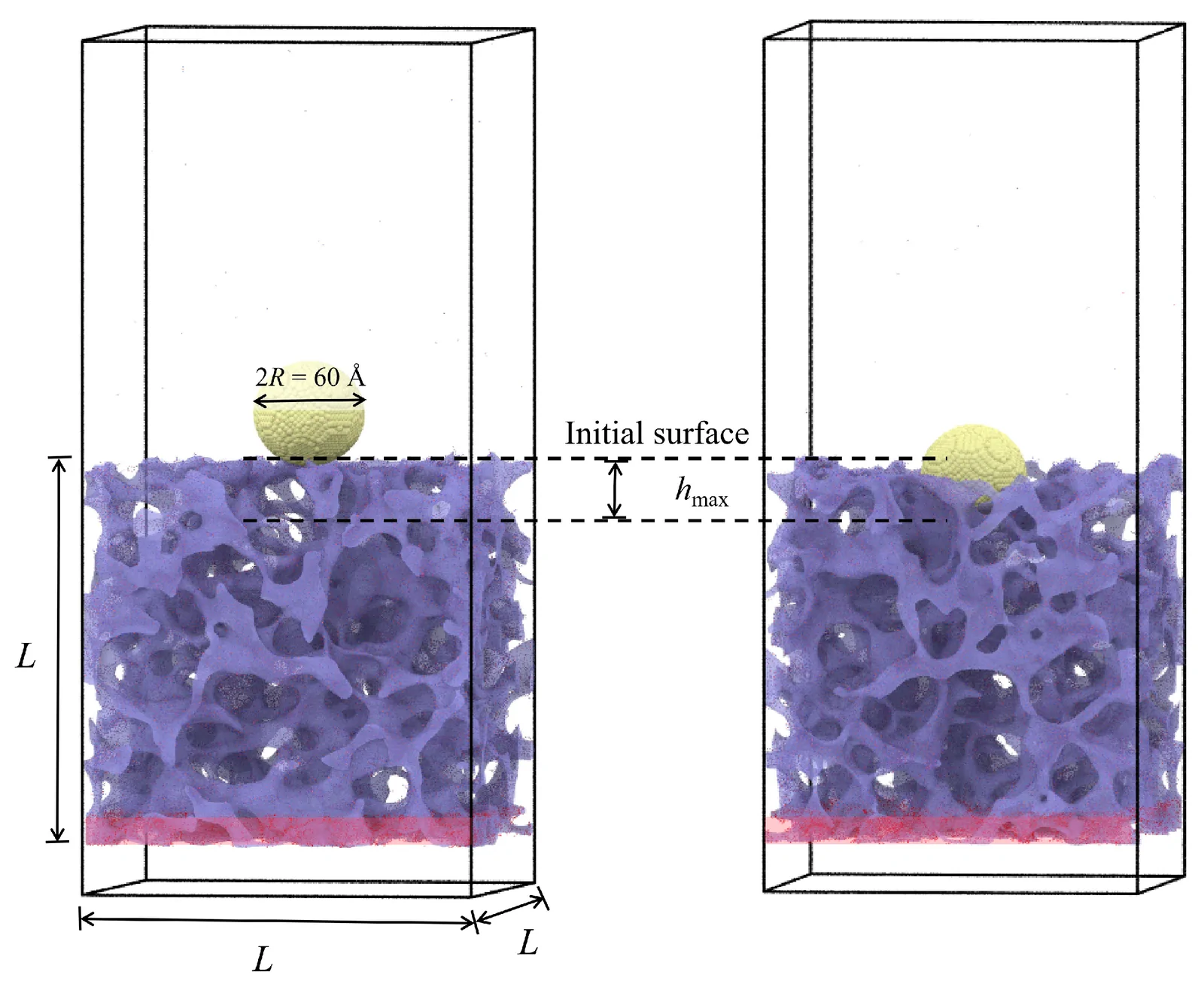
Schematic of the nanoindentation molecular dynamics simulation system.

The nanoindentation simulation model is shown in Figure 14. The rigid indenter was constructed according to a diamond lattice as a hollow sphere with an outer radius of 30 Å and an inner radius of 28 Å, initially hovering 2 Å above the matrix. The red region at the bottom of the model was set as a fixed boundary layer. The non-bonded interactions between the indenter and the matrix were described using the Lennard–Jones potential with a cutoff radius of 10 Å. The energy and size parameters for silicon and carbon atoms were 0.008909 eV and 3.326 Å, respectively, while those for oxygen and carbon atoms were 0.003442 eV and 3.001 Å [59].

The indentation cycle consisted of three phases: constant-rate loading, holding, and unloading. The indenter was pressed downward along the Z-axis into the matrix at a rate of 0.1 Å/ps to the target depth and remained stationary there for 20 ps. Subsequently, the indenter was retracted upward at the same rate to its initial height. The load–displacement response and atomic motion trajectories throughout the entire indentation process were completely recorded and output, providing the data foundation for subsequent calculations of local effective mechanical indicators and analysis of pore topological evolution mechanisms. In this study, the rigid spherical indenter serves as a local processing tool that generates a controllable non-uniform stress field to induce localized densification, rather than as a model of the in-service mechanical loading of a flexible device. The spherical geometry is a standard configuration in the nanoindentation of silica aerogels and facilitates the extraction of local effective mechanical parameters within the Hertz/Oliver–Pharr framework, while the rigid assumption confines the analysis to the response of the aerogel matrix itself [34,45]. Varying the indentation depth (50–150 Å) systematically tunes the local stress level and the degree of densification.

### 4.3. Calculation of Elastic Modulus and Hardness of Aerogels

**Figure 15:**
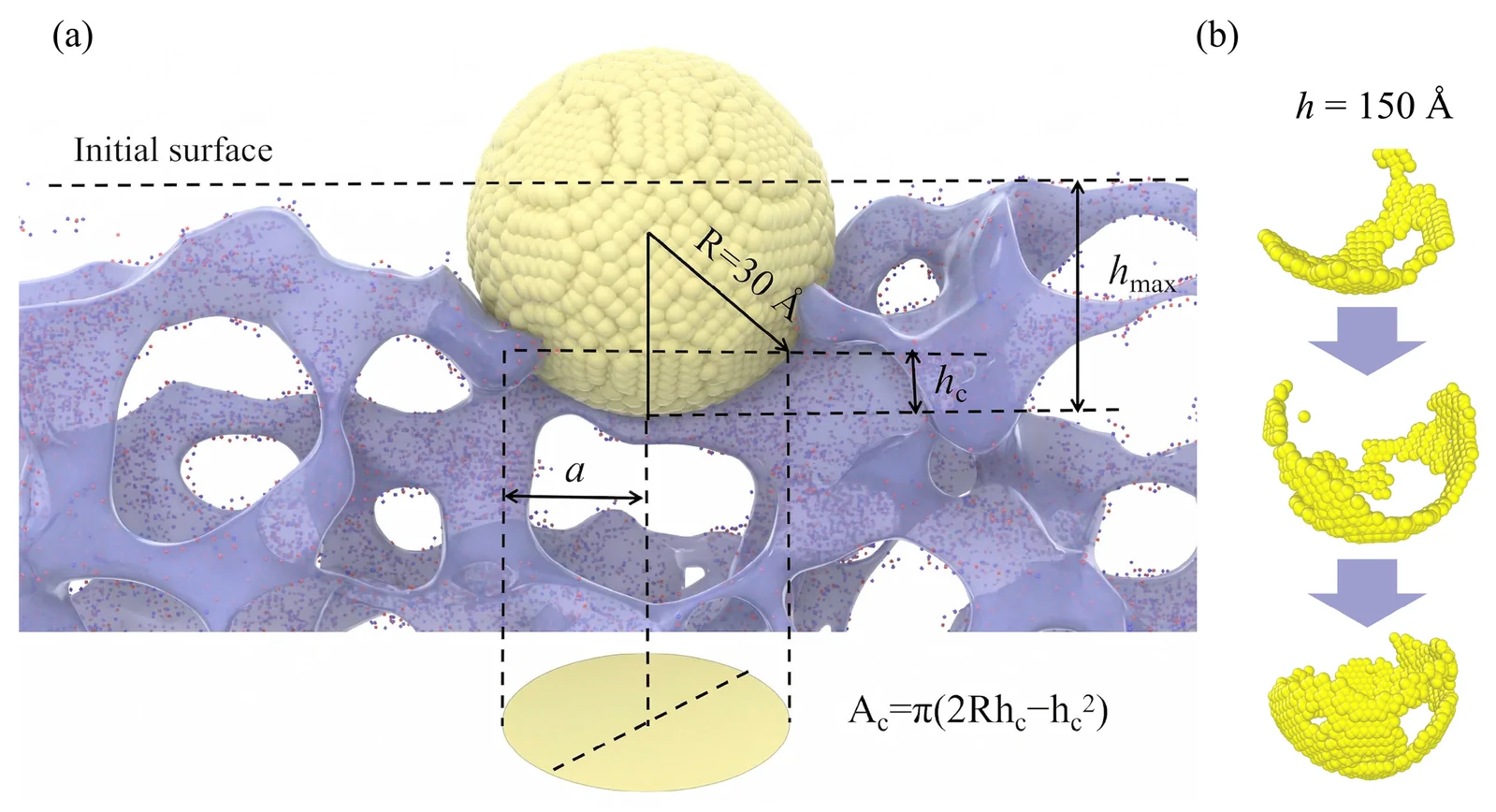
Contact mechanics model and area evolution during the nanoindentation process. (**a**) Schematic of spatial geometric projection and effective contact area calculation during the indentation process; (**b**) Morphological evolution of the effective contact region between the aerogel framework and the indenter as the indentation depth reaches 150 Å.

The elastic modulus and hardness of the aerogel matrix are extracted from the unloading segment of the load–displacement curve during the shallow nanoindentation stage using the Oliver–Pharr method [34], and its core spatial geometric projection relationship is illustrated in Figure 15a. During the nanoindentation process, the actual contact morphology between the indenter and the porous silica framework undergoes dynamic evolution as the indentation depth increases. Taking the maximum indentation depth of 150 Å at 298 K as an example, Figure 15b extracts the three-dimensional (3D) morphology of the effective contact region at three typical evolution moments (400 ps, 800 ps, and 1200 ps) during the loading phase. As observed from top to bottom in the figure, as the indenter continuously advances downward, the aerogel framework undergoes pore collapse and network rearrangement due to localized compression; consequently, its actual physical contact region gradually expands and densifies from the initial localized discrete contact into a continuous hemispherical enveloping structure. This dynamic evolution of the microscopic contact area provides a physical basis for the subsequent accurate extraction of macroscopic mechanical parameters.

By fitting the initial linear segment of the unloading curve, the contact stiffness at the onset of unloading, $S={\left.\frac{\mathrm{dP}}{\mathrm{dh}}\right|}_{\max}$, can be accurately obtained. For the spherical indenter used in this study, the contact depth $h_{c}$ is given by the following equation:

$$h_{c}=h_{\max}-\varepsilon \frac{P_{\max}}{S}$$

where $h_{\max}$ is the maximum indentation depth, $P_{\max}$ is the peak load, and ϵ=0.75 is the geometric constant for the spherical indenter. From this, the projected contact area $A_{c}$ can be derived:

$$A_{c}=\pi (2Rh_{c}-h_{c}^{2})$$

where R=30 Å is the indenter radius. This area is physically equivalent to the effective circular contact plane with radius a shown in Figure 15. The reduced modulus $E_{r}$ can be further derived from the contact stiffness and contact area:

$$E_{r}=\frac{\sqrt{\pi }}{2\beta }\frac{S}{\sqrt{A_{c}}}$$

where β=1.034 is the indenter shape correction factor. Subsequently, the true elastic modulus E of the sample can be obtained by the following equation:

$$\frac{1}{E_{r}}=\frac{1-\nu^{2}}{E}+\frac{1-\nu_{i}^{2}}{E_{i}}$$

Here, $E_{i}=1141\;\mathrm{GPa}$ and $\nu_{i}=0.07$ are the elastic modulus and Poisson’s ratio of the diamond indenter, respectively [60], and ν is the Poisson’s ratio of the silica aerogel. Meanwhile, the hardness H of the material is defined as the ratio of the peak load to the projected contact area:

$$H=\frac{P_{\max}}{A_{c}}$$

It should be noted that, due to the high porosity of aerogels and pore collapse during unloading, the Oliver–Pharr method in this study yields an equivalent or apparent response of the local region beneath the indenter, which is not equivalent to the bulk macroscopic modulus or macroscopic yield strength. Therefore, the reported modulus and hardness are used as relative indicators of local mechanical response rather than absolute material constants.

### 4.4. Pore Structure Characterization Algorithm

Regarding the extraction of the pore size distribution, the probe sphere method was employed in this study to traverse the free volume within the amorphous silica framework to identify all accessible pore spaces [61]. In the analysis, the probe radius was set to 2.0 Å, the minimum identifiable pore radius threshold was 1.0 Å, and three-dimensional (3D) periodic boundary conditions were considered. This method can output the number density distribution histograms of pore radii at various evolution moments, thereby tracking the shrinkage behavior of large-sized pores and the generation and disappearance rules of small-sized pores during the localized densification process of the material under compression.

**Figure 16:**
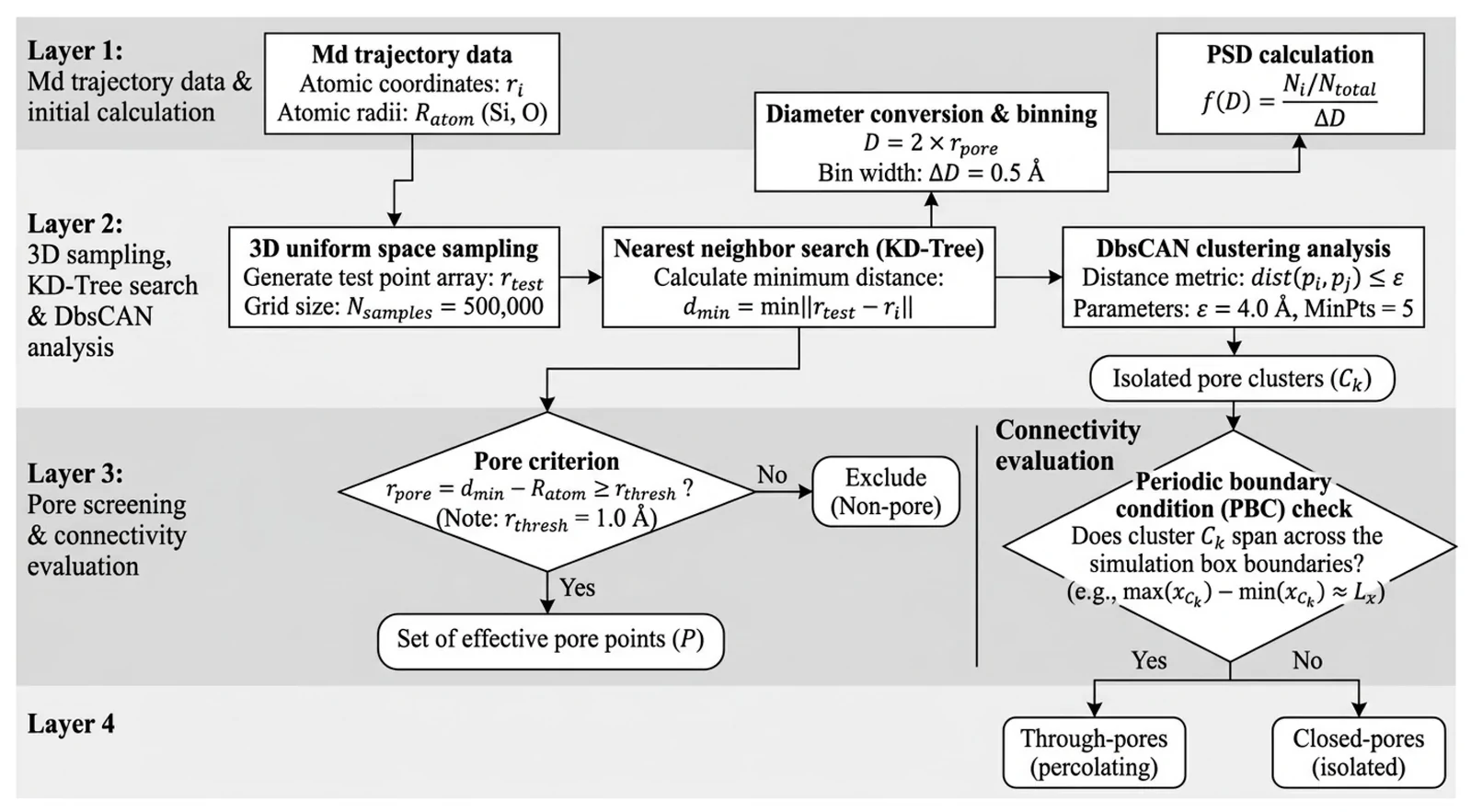
Flowchart of the pore structure characterization algorithm.

The determination of pore connectivity is based on clustering analysis [62]. When the distance between two pore centers is less than the clustering distance threshold of 4.0 Å, they are considered to belong to the same pore cluster. Based on the crossing behavior of the clusters under periodic boundary conditions, connected pore clusters are defined as clusters that span across the simulation box boundaries in at least one direction, while isolated pore clusters are defined as clusters that are completely isolated in all directions. The analysis simultaneously records the number of isolated pore points as a supplementary reference. Figure 16 illustrates the flowchart of the pore structure characterization algorithm in this study. The aforementioned pore structure characterizations are conducted through systematic post-processing of the trajectory files throughout the entire indentation process, thereby obtaining the evolution data of pore size distribution and connectivity at different moments, which provides a quantitative topological basis for revealing the sintering mechanisms under the synergistic effect of temperature and indentation depth.

## Figures and Tables

**Table 1:** Simulation parameters and their sources.

Parameter	Value	Source
Potential for Si–O interactions	Tersoff potential	[57]
Potential for indenter–matrix interactions	Lennard–Jones potential, cutoff 10 Å	[59]
Si–C LJ parameters	ε = 0.008909 eV, σ = 3.326 Å	[59]
O–C LJ parameters	ε = 0.003442 eV, σ = 3.001 Å	[59]
Aerogel density	710 kg/m^3^	[35,36,37,38,39,41,42,43,44]
Porosity	67.7%	—
Box size	230 × 230 × 230 Å^3^	—
Number of atoms	~192,000 (silica) + ~3700 (indenter)	[43,44]
Thermostat	Nose–Hoover, NVT ensemble	—
Timestep	0.001 ps	—
Boundary conditions	p p p	—
Fixed layer thickness	15 Å	[58]
Temperature range	298–1800 K	[25]
Indentation depths	50, 80, 100, 120, 150 Å	—
Loading/unloading rate	0.1 Å/ps	[34]
Holding time at max depth	20 ps	—
Number of independent cases	35 (5 depths × 7 temperatures)	—
Indenter geometry	Hollow sphere, R_outer = 30 Å, R_inner = 28 Å	[34]
Indenter material	Rigid diamond-like carbon	[34]

## Data Availability

The data presented in this study are available on request from the corresponding author.

## Supplementary Materials

The following supporting information can be downloaded at: https://www.mdpi.com/article/10.3390/gels12080684/s1. Figures S1–S5: Load-displacement curves of the silica-inspired aerogel model at indentation depths of 50, 80, 100, 120, and 150 Å. Figures S6–S10: Evolution of pore size distribution at different temperatures and time points for indentation depths of 50, 80, 100, 120, and 150 Å. Figures S11–S13: Evolution of pore connectivity over time at intermediate indentation depths of 80, 100, and 120 Å. Figures S14–S19: Multi-depth comparison of pore connectivity at 298, 450, 600, 973, 1500, and 1800 K. Videos S1–S4: Dynamic atomistic visualizations of the full nanoindentation process at a depth of 150 Å under 298, 973, 1300, and 1500 K. Video S5: Time-resolved 3D spatial evolution of the connected pore clusters at 1300 K (150 Å depth). Video S6: Time-resolved 3D spatial evolution of the isolated pore clusters at 1300 K (150 Å depth).
