# Supplementary material for: Silica-Inspired Aerogel Thermal Metamaterials with Gradient Porosity: High-Temperature-Induced Pore Sintering Evolution via Nanoindentation
Source: Gels. 2026 Aug 3;12(8):684. doi: 10.3390/gels12080684 (PMC13512897; doi:10.3390/gels12080684)
Supplement: Supplementary file 1 [file gels-12-00684-s001.zip › Supporting Information_gels.pdf]

Supplementary Materials

Silica-Inspired Aerogel Thermal Metamaterials with Gradient Porosity: High-Temperature-Induced Pore Sintering Evolution via Nanoindentation

Yiming Song [1], Mingyang Yang [1,2,*], Shuxu Li [1], Huiyu Yang [1], Ying Yin [3] and Mu Du [4,5]

[1] School of Resources Engineering, Xi'an University of Architecture and Technology, Xi'an 710055, China
[2] Key Laboratory of Thermo-Fluid Science and Engineering (Xi'an Jiaotong University), Ministry of Education, Xi'an 710049, China
[3] Shandong Key Laboratory of Hydrogen Energy Equipment and Safety, College of New Energy, China University of Petroleum (East China), Qingdao 266580, China
[4] Shenzhen Research Institute, Shandong University, Shenzhen 518057, China
[5] Institute for Advanced Technology, Shandong University, Jinan 250061, China
* Correspondence: myyang@xauat.edu.cn

Section S1. Load-displacement curves of aerogels

Figures S1 to S5 present the comprehensive load-displacement curves of silica aerogels during the nanoindentation process under varying environmental temperatures (298 K to 1500 K) and specific indentation depths (50, 80, 100, 120, and 150 Å). These supplementary parametric studies complement Section 3.1 in the main text, confirming the consistent occurrence of the anomalous load-bearing enhancement behavior at extreme temperatures across multiple depths.

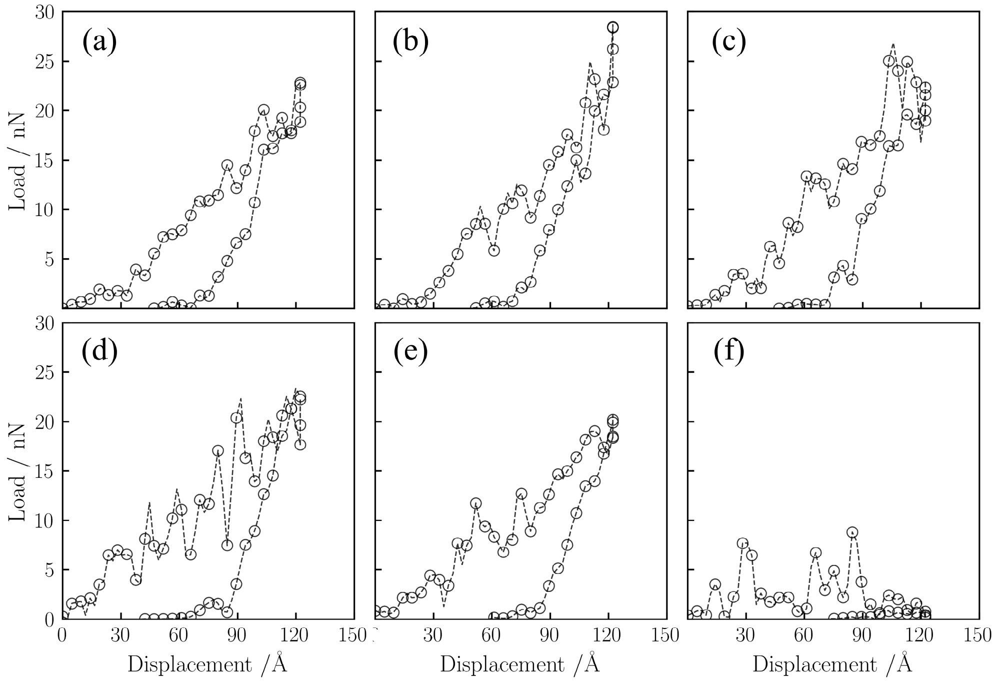

Figure S1. Load-displacement curves of aerogels at various temperatures for an indentation depth of 50 Å. (a) 298 K; (b) 450 K; (c) 600 K; (d) 973 K; (e) 1300 K; (f) 1500 K.

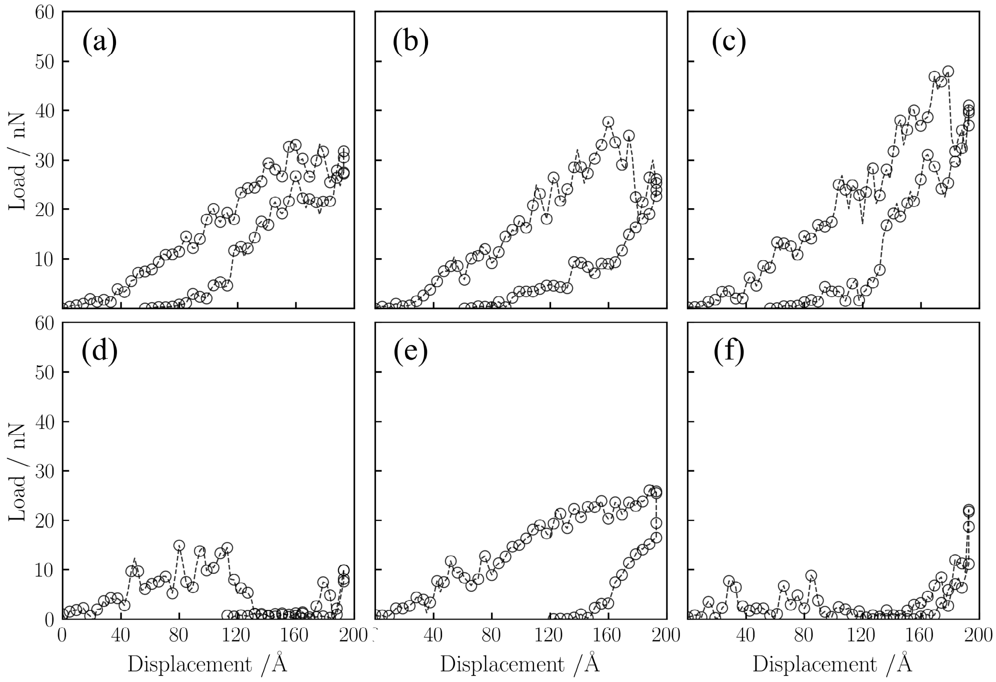

Figure S2. Load-displacement curves of aerogels at various temperatures for an indentation depth of 80 Å. (a) 298 K; (b) 450 K; (c) 600 K; (d) 973 K; (e) 1300 K; (f) 1500 K.

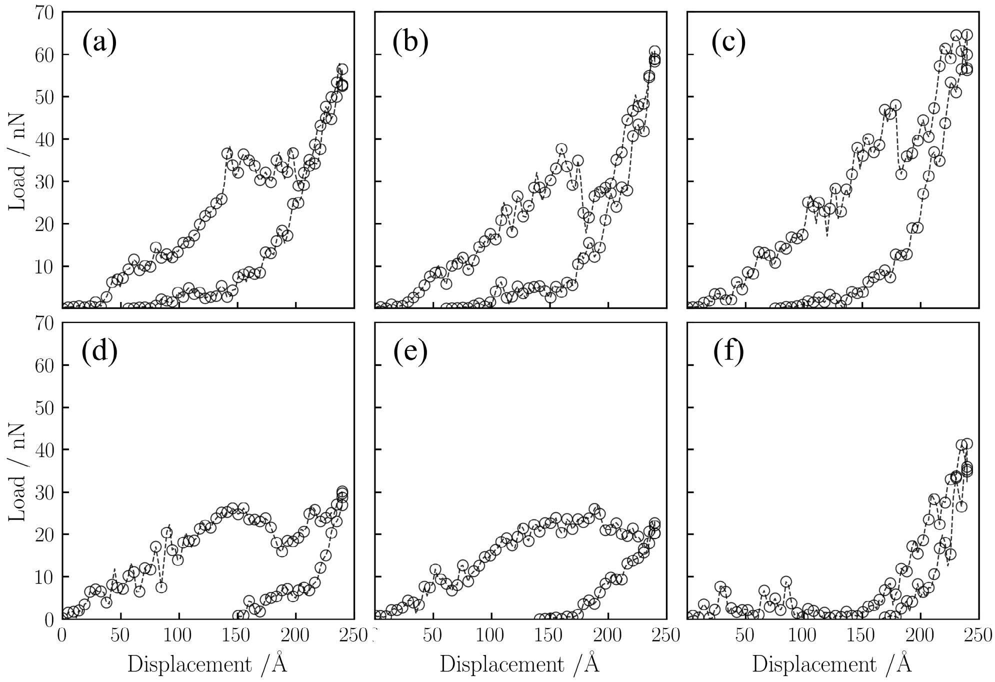

Figure S3. Load-displacement curves of aerogels at various temperatures for an indentation depth of 100 Å. (a) 298 K; (b) 450 K; (c) 600 K; (d) 973 K; (e) 1300 K; (f) 1500 K.

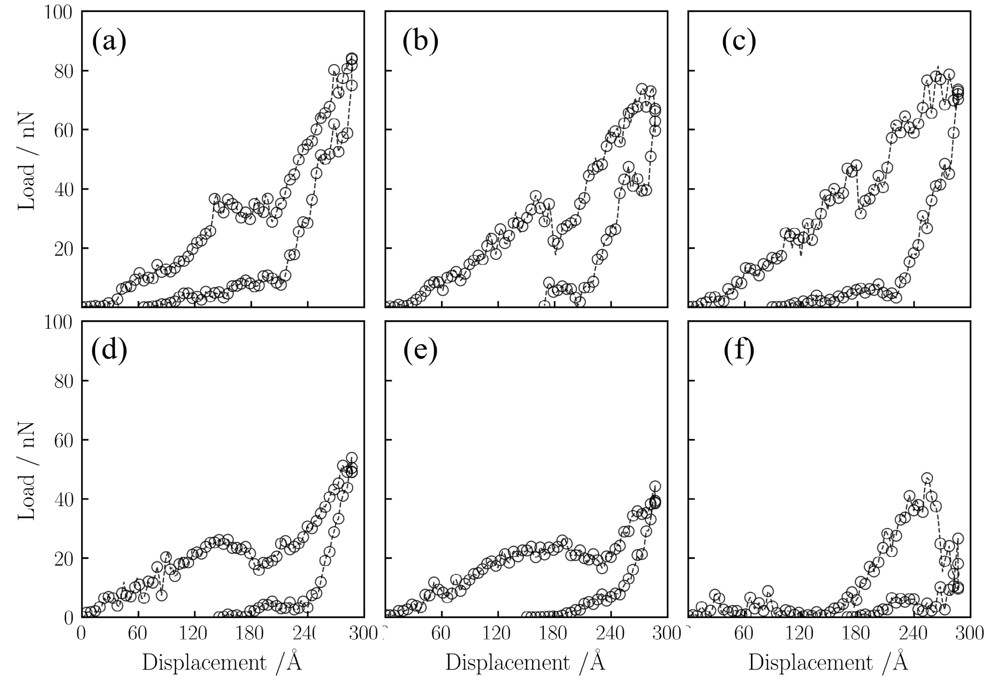

Figure S4. Load-displacement curves of aerogels at various temperatures for an indentation depth of 120 Å. (a) 298 K; (b) 450 K; (c) 600 K; (d) 973 K; (e) 1300 K; (f) 1500 K.

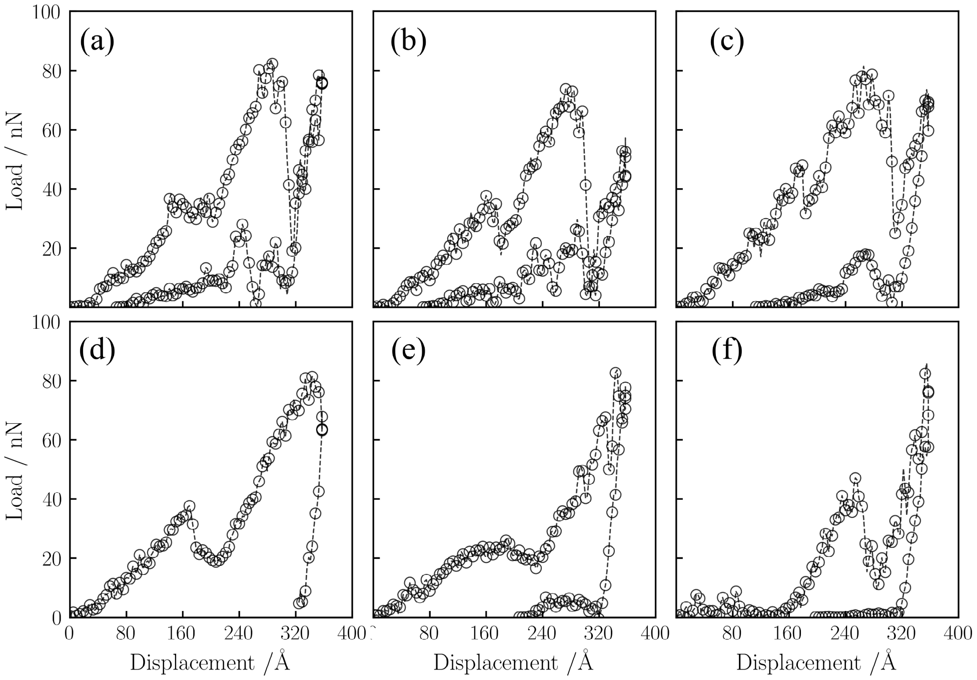

Figure S5. Load-displacement curves of aerogels at various temperatures for an indentation depth of 150 Å. (a) 298 K; (b) 450 K; (c) 600 K; (d) 973 K; (e) 1300 K; (f) 1500 K.

Section S2. Detailed evolution of pore size distribution

Figures S6 to S10 provide the detailed microstructural evolution of the pore size distribution across all simulated depths and temperatures. These results supplement Section 4.1, further validating the pore-size dependence of the sintering process and providing systematic evidence for the transition from bimodal splitting to isolated densification.

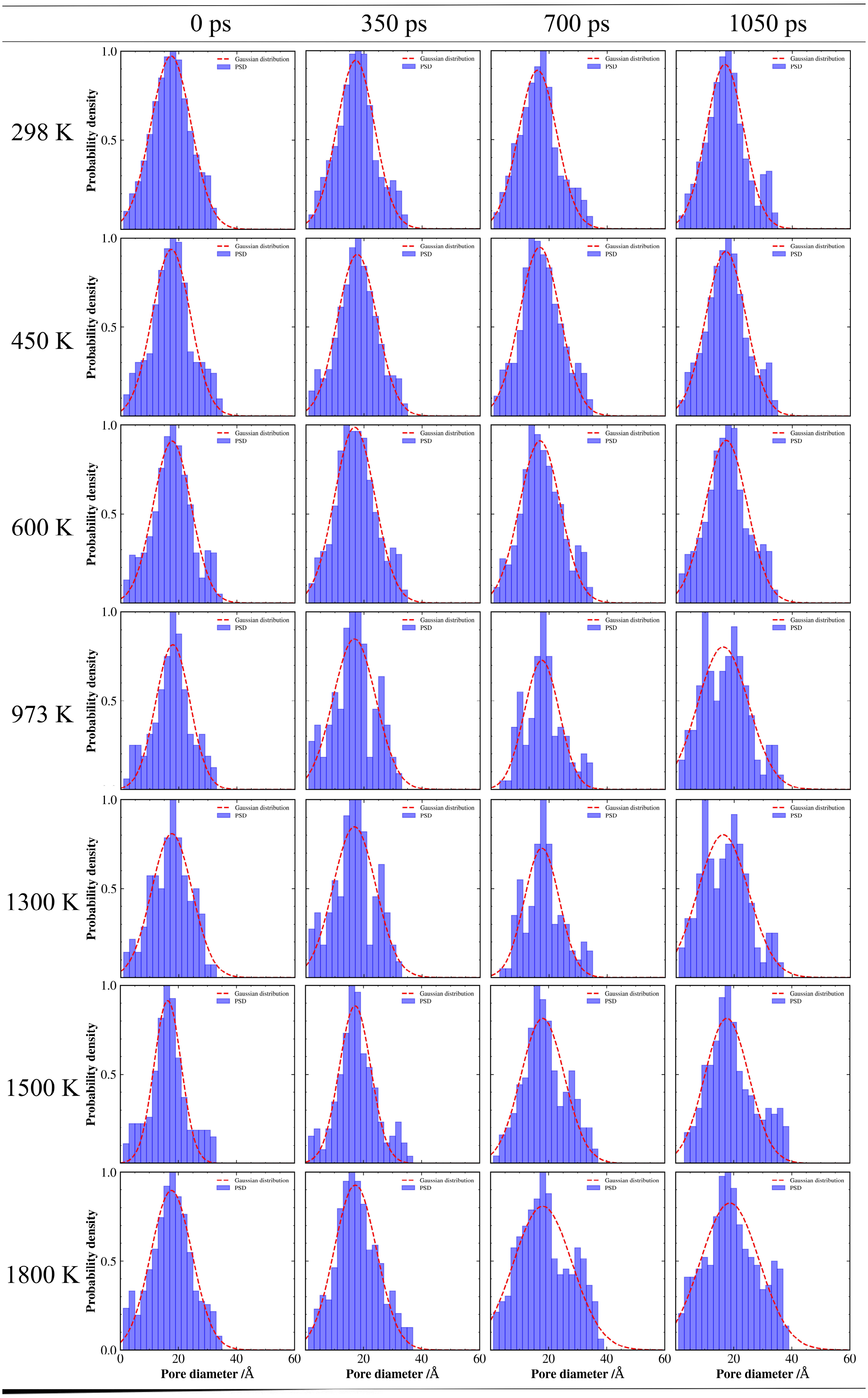

Figure S6. Evolution of pore size distribution at different temperatures and time points for an indentation depth of 50 Å

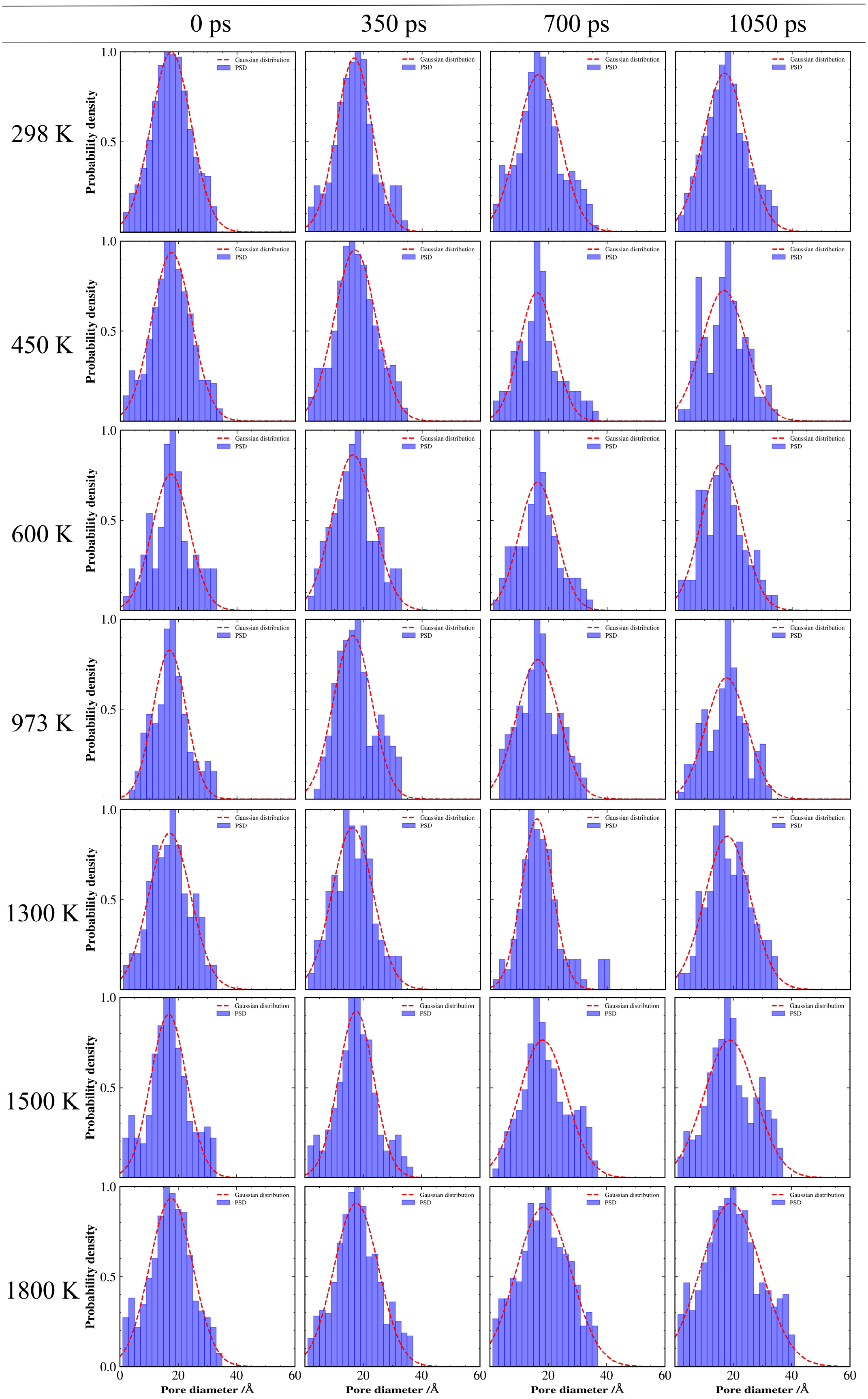

Figure S7. Evolution of pore size distribution at different temperatures and time points for an indentation depth of 80 Å.

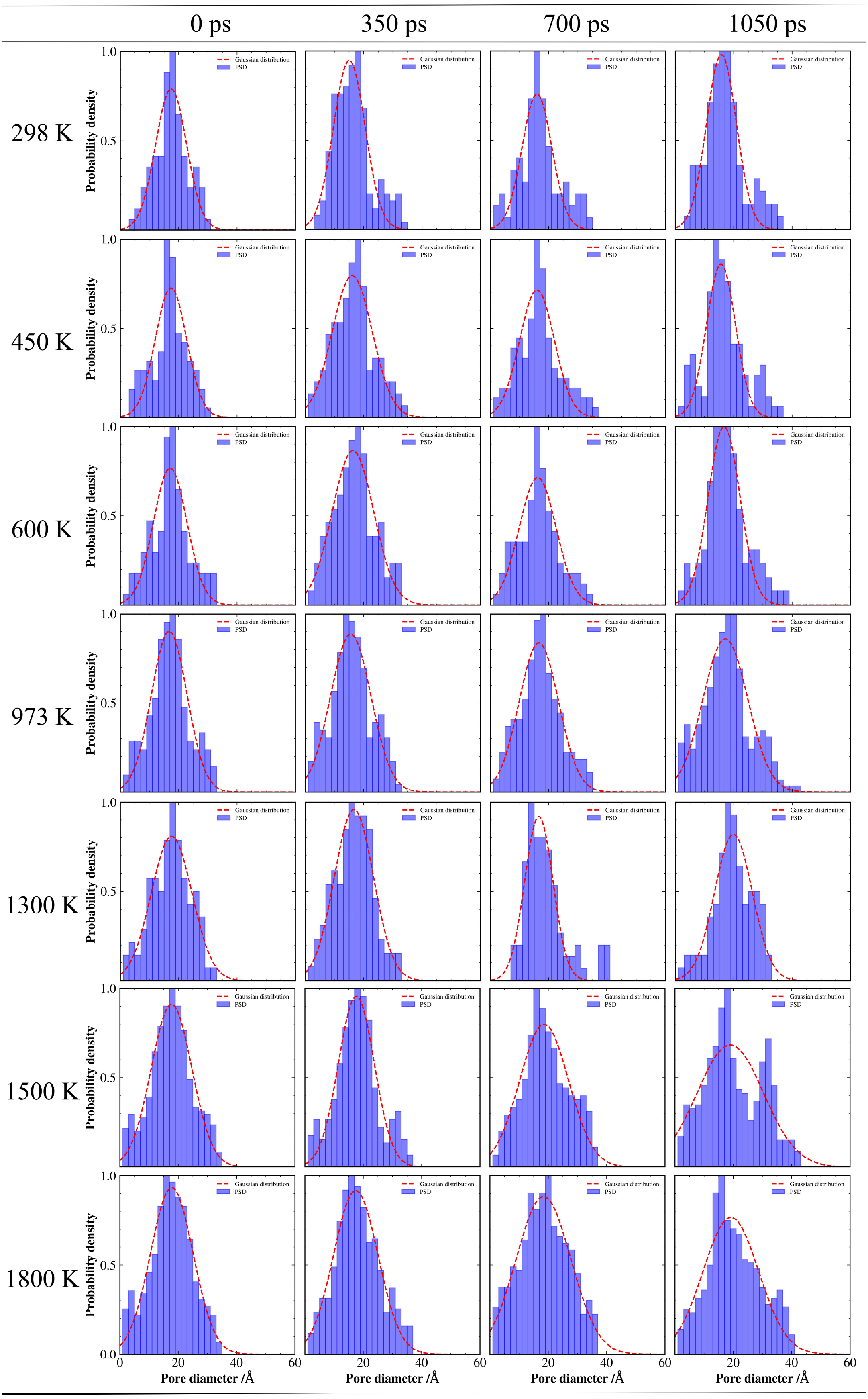

Figure S8. Evolution of pore size distribution at different temperatures and time points for an indentation depth of 100 Å.

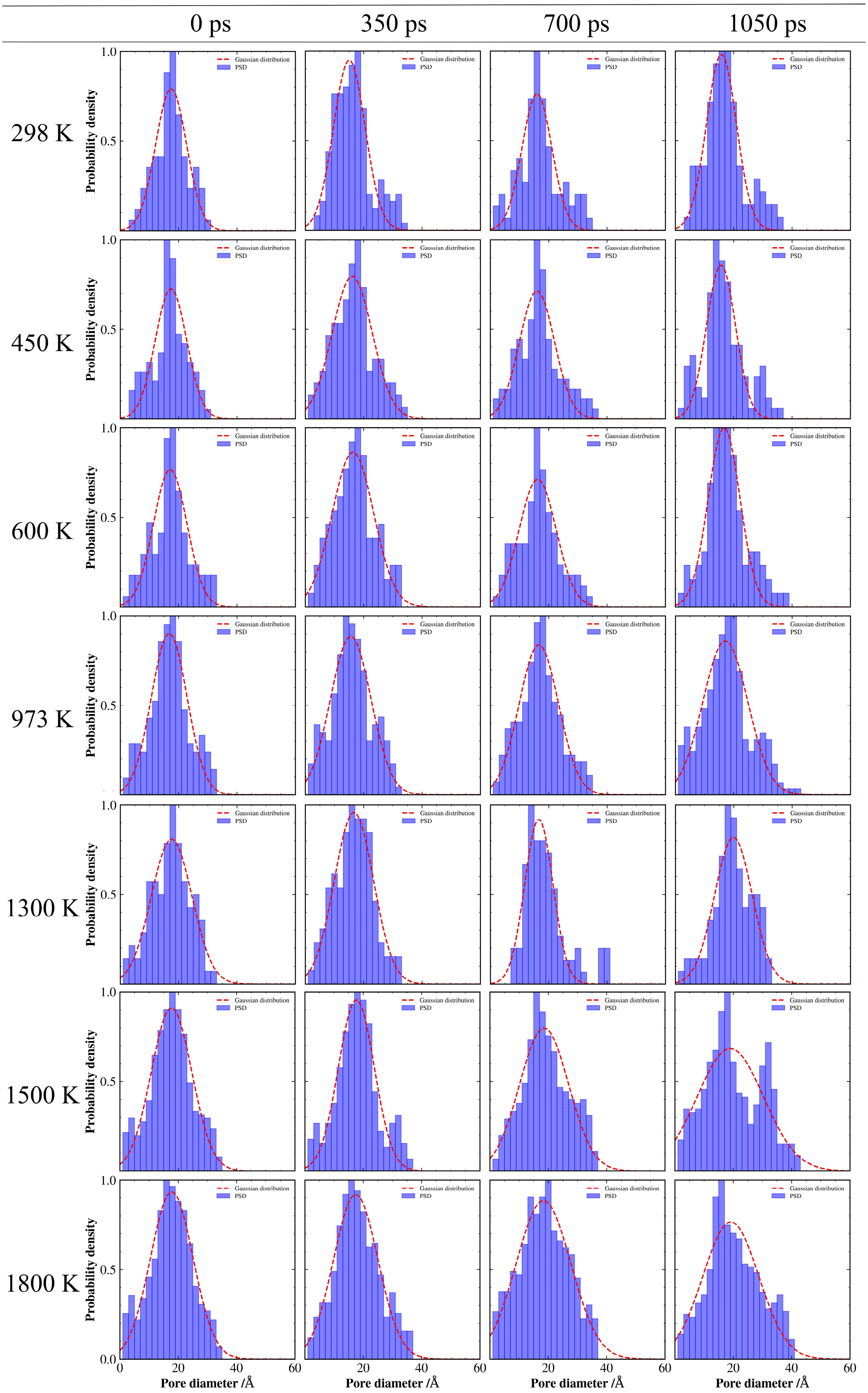

Figure S9. Evolution of pore size distribution at different temperatures and time points for an indentation depth of 120 Å.

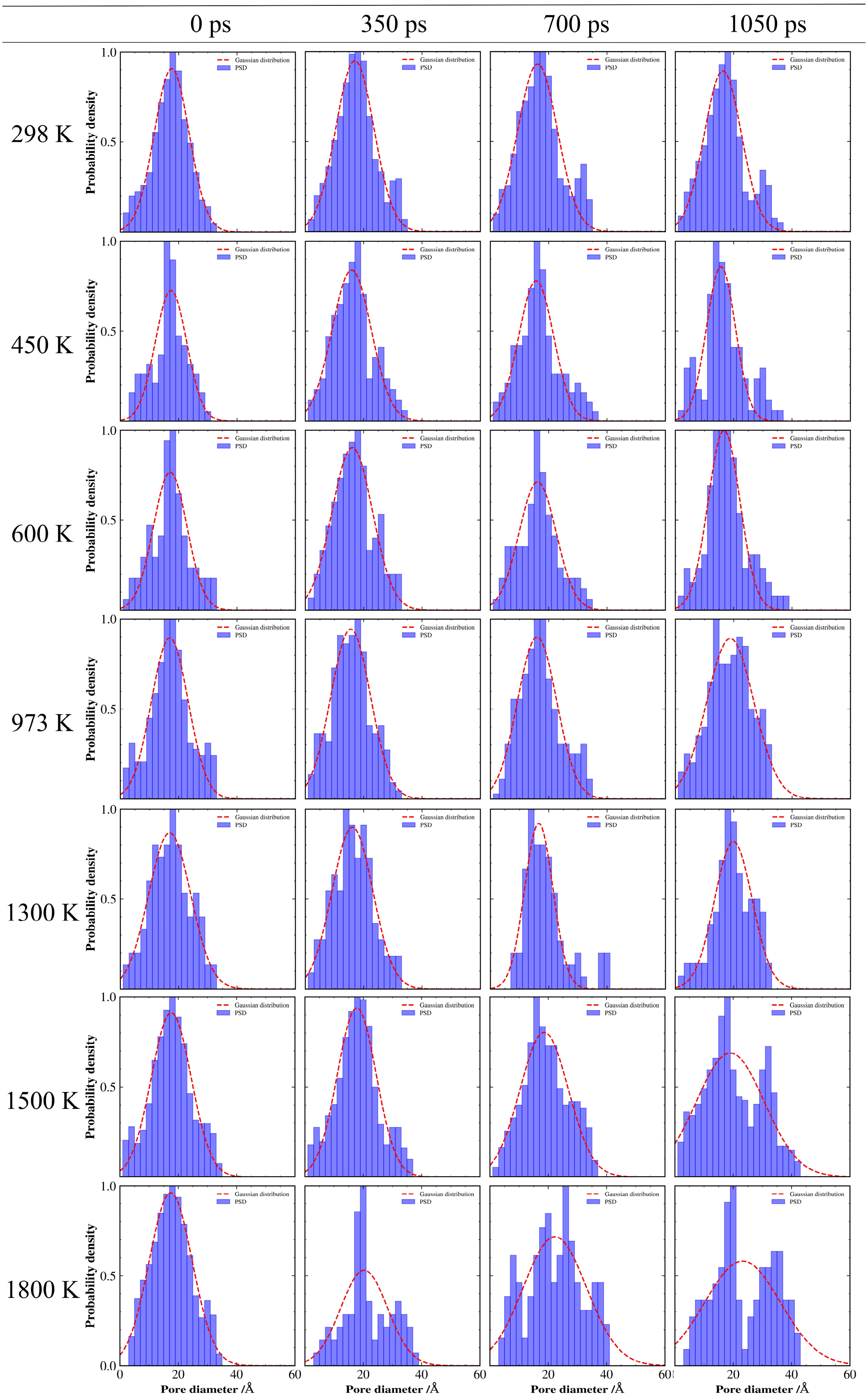

Figure S10. Evolution of pore size distribution at different temperatures and time points for an indentation depth of 150 Å.

Section S3. Evolution of pore connectivity over time

Figures S11 to S13 expand the analysis of pore connectivity presented in Section 4.2 to the intermediate indentation depths (80, 100, and 120 Å). The data quantitatively tracks the fracture of connected pore clusters and the aggregation of isolated pores, demonstrating the continuous topological phase transition across varying local stress states.

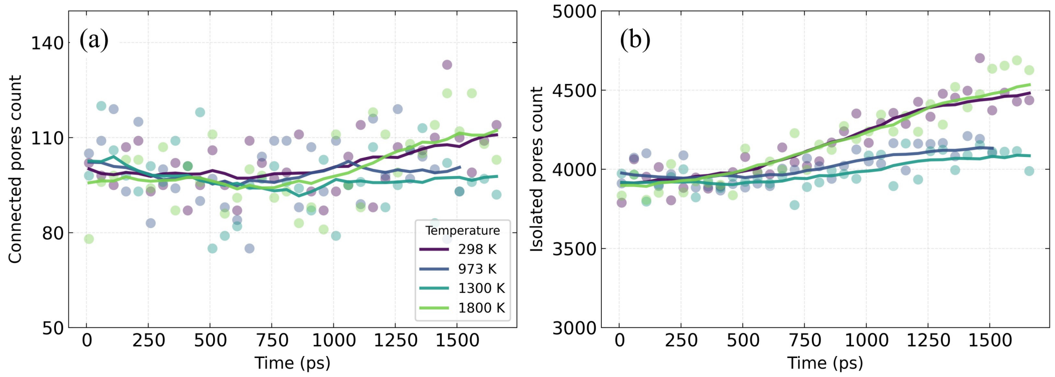

Figure S11. Evolution of pore connectivity over time at an indentation depth of 80 Å. (a) Evolution of connected pore clusters; (b) Evolution of isolated pore clusters.

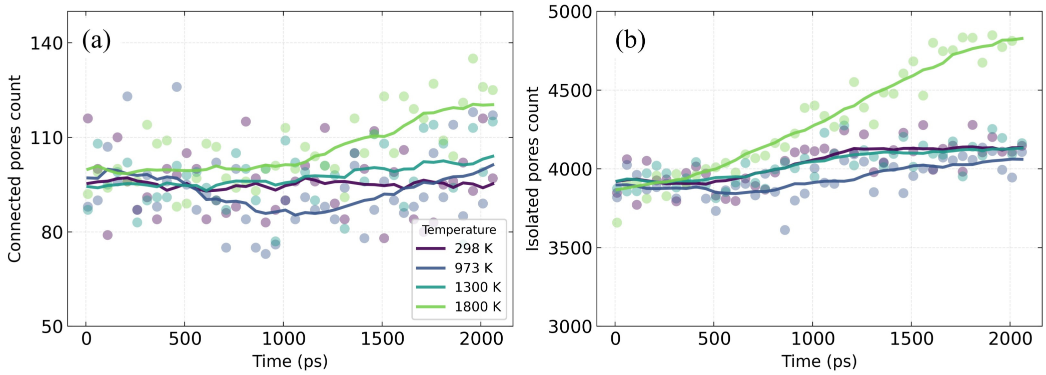

Figure S12. Evolution of pore connectivity over time at an indentation depth of 100 Å. (a) Evolution of connected pore clusters; (b) Evolution of isolated pore clusters.

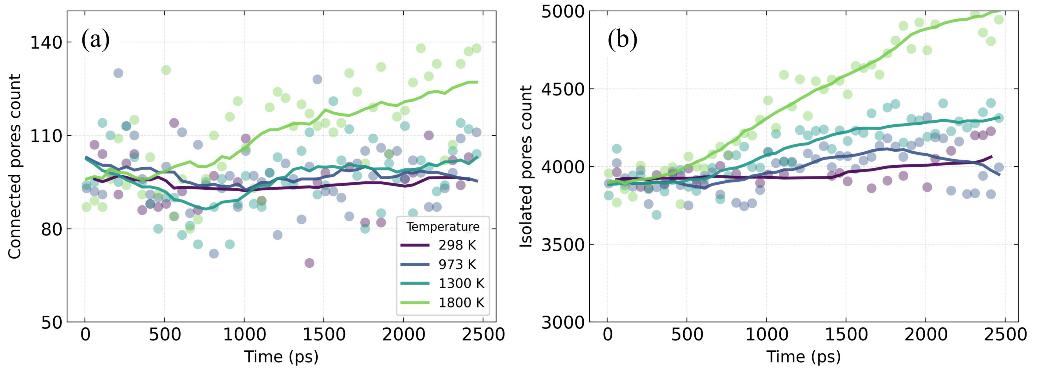

Figure S13. Evolution of pore connectivity over time at an indentation depth of 120 Å. (a) Evolution of connected pore clusters; (b) Evolution of isolated pore clusters.

Section S4. Multi-depth comparison of pore connectivity

Figures S14 to S19 present a comprehensive multi-depth comparative analysis of pore cluster evolution under varying temperature conditions (298 K, 450 K, 600 K, 973 K, 1500 K, and 1800 K). These macroscopic topological mappings thoroughly support the conclusions in Section 4.3, visually confirming the spatial acceleration effect of localized indentation stress on the structural densification timeline.

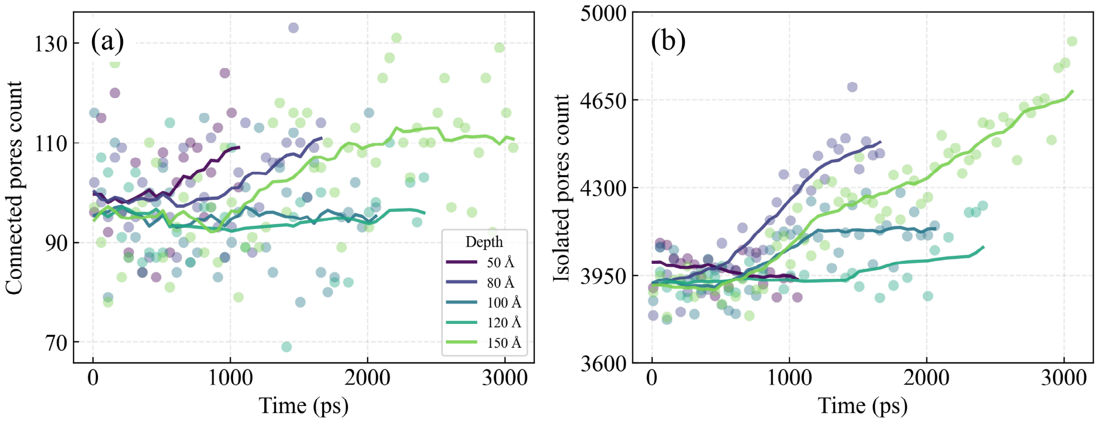

Figure S14. Multi-depth comparison of pore connectivity at 298 K. (a) Evolution of connected pore clusters over time; (b) Evolution of isolated pore clusters over time.

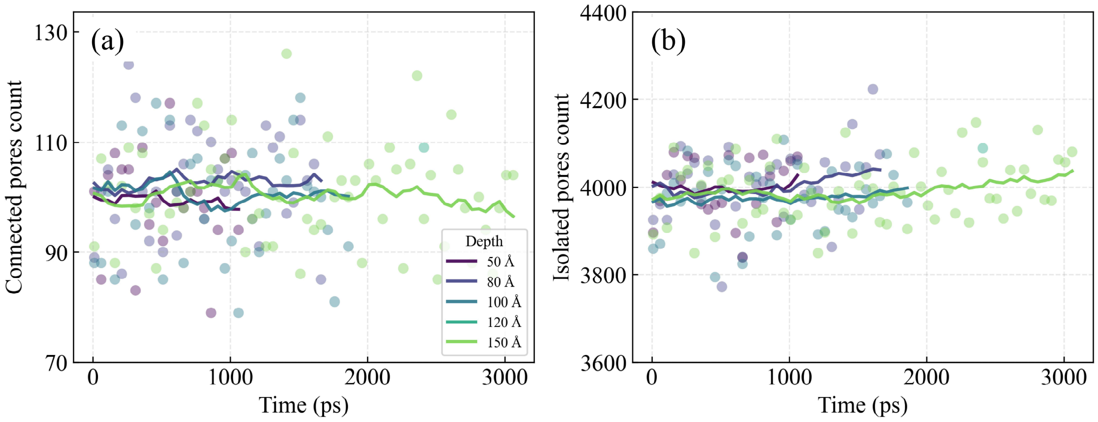

Figure S15. Multi-depth comparison of pore connectivity at 450 K. (a) Evolution of connected pore clusters over time; (b) Evolution of isolated pore clusters over time.

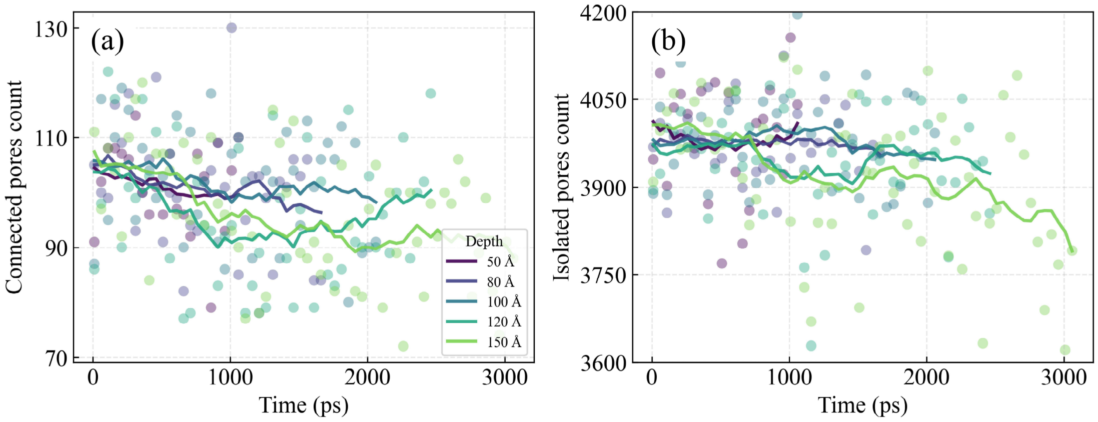

Figure S16. Multi-depth comparison of pore connectivity at 600 K. (a) Evolution of connected

pore clusters over time; (b) Evolution of isolated pore clusters over time.

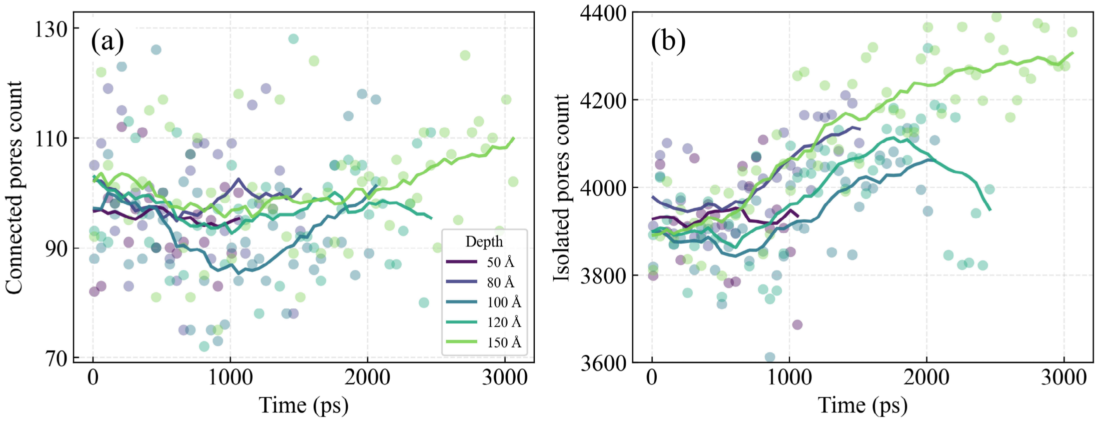

Figure S17. Multi-depth comparison of pore connectivity at 973 K. (a) Evolution of connected pore clusters over time; (b) Evolution of isolated pore clusters over time.

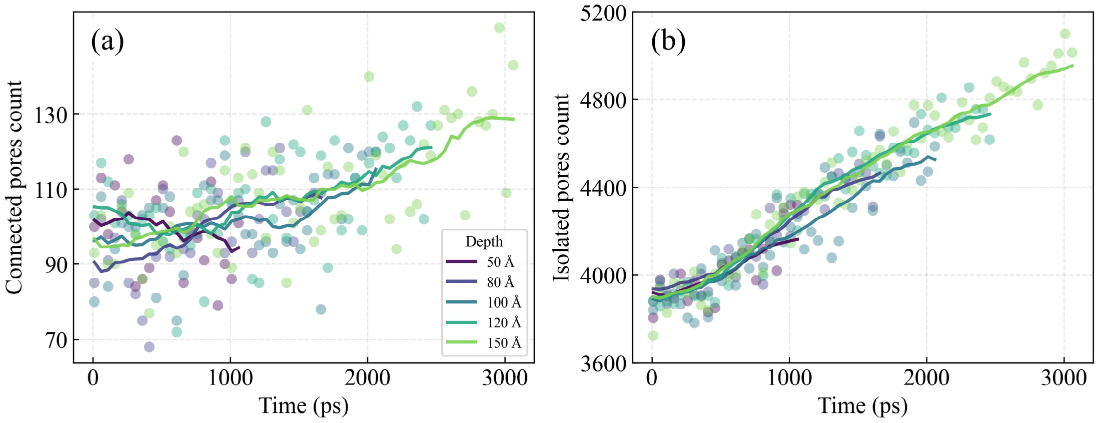

Figure S18. Multi-depth comparison of pore connectivity at 1500 K. (a) Evolution of connected pore clusters over time; (b) Evolution of isolated pore clusters over time.

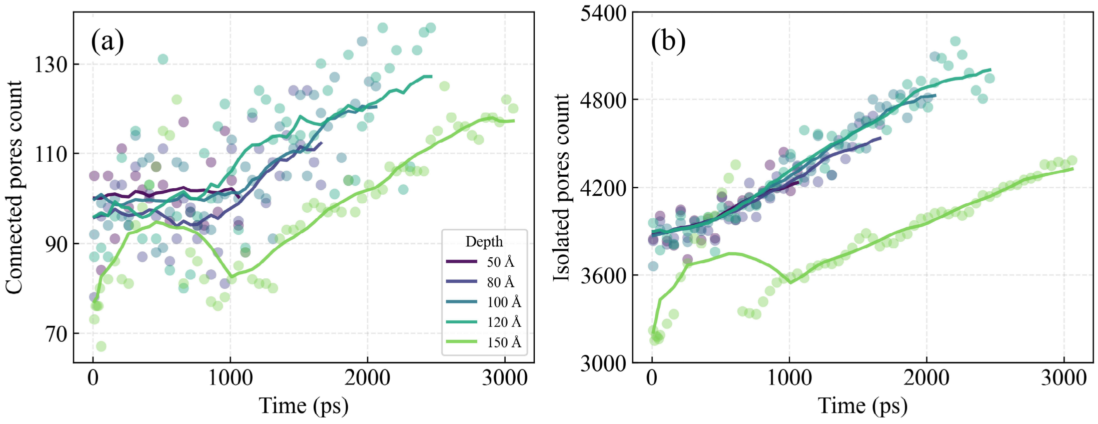

Figure S19. Multi-depth comparison of pore connectivity at 1800 K. (a) Evolution of connected pore clusters over time; (b) Evolution of isolated pore clusters over time.

Section S5. Supplementary Videos

Video S1-S4. Dynamic atomistic visualizations of the full nanoindentation process at a depth of 150 Å under varying temperatures (298 K, 973 K, 1300 K, and 1500 K).

Video S5. Time-resolved 3D spatial evolution of the connected pore clusters at 1300 K (150 Å depth).

Video S6. Time-resolved 3D spatial evolution of the isolated pore clusters at 1300 K (150 Å depth).
